# Unraveling the Influence of Behavioural Ecotypes on Fish Gut Microbiome: Focus on the Atlantic cod (*Gadus morhua*) in Icelandic Waters

**DOI:** 10.1007/s00248-026-02832-0

**Published:** 2026-07-15

**Authors:** Elena Radaelli, Giorgia Palladino, Daniela Leuzzi, Daniel Scicchitano, Simone Rampelli, Silvia Turroni, Haseeb S. Randhawa, Marco Candela

**Affiliations:** 1 Department of Pharmacy and Biotechnology (FaBit), Unit of Microbiome Science and Biotechnology, Alma Mater Studiorum–University of Bologna, Bologna, Italy; 2 Fano Marine Center, Resources and Biotechnology, the Inter–institute Center for Research on Marine Biodiversity, Fano, Italy; 3 Faculty of Life and Environmental Sciences, University of Iceland, Reykjavík, Iceland; 4 New Brunswick Museum, Saint John, New Brunswick Canada

**Keywords:** Atlantic cod, Iceland, Ecotypes, Microbiome, Metabarcoding

## Abstract

This study investigated the gut microbiome of Atlantic cod in northern Icelandic waters to assess how resident (coastal) and migratory (frontal) behavioural ecotypes influence gut microbial community composition, with a focus on the effects of ecological adaptation and the potential drivers of microbial shifts within this species. A total of 81 intestinal samples from coastal and frontal Atlantic cod collected in northern Iceland in December 2021 and January 2024 were analysed using 16S rRNA gene metabarcoding. Environmental datasets from the Copernicus Marine Service were used to assess the relationship between annual shifts in microbiome composition and changes in environmental conditions. Our results highlight that the Icelandic cod gut microbiome is a highly dynamic system primarily shaped by environmental shifts, such as warming trends and anthropogenic stressors. Because different behavioural ecotypes are inherently exposed to distinct environmental configurations, these distinct macro–scale exposures may indirectly translate into ecotype–specific microbial signatures that shape the differentiation between coastal and offshore habitats; the effects were particularly evident in the coastal populations, underscoring the greater vulnerability of nearshore habitats to environmental shifts and anthropogenic stressors. These findings suggest the role of the microbiome in ecological plasticity of Atlantic cod; concurrently, they reveal distinct ecotype–specific sensitivities to environmental shifts, particularly those of anthropogenic origin, providing a valuable framework that may support future marine conservation strategies.

**Supplementary Information:**

The online version contains supplementary material available at https://doi.org/10.1007/s00248-026-02832-0.

## Introduction

Atlantic cod (*Gadus morhua*) is one of the most commercially and culturally important marine fish species, representing a major economic resource in several countries across northern Europe and along the eastern coast of North America [32]. Distributed across vast regions of the Atlantic Ocean, this omnivorous species exhibits high fecundity and marked local adaptations in phenotype, behaviour, and genetics, including differences in growth rate, age at maturity, migration patterns, and reproduction times [11, 49]. Local adaptations in Atlantic cod have evolved in relation to both geographic distribution [36] and behavioural patterns (Sinclair–Waters et al. [63, 79], and are mainly maintained between populations by differences in behaviour and genetic divergence [5, 6, 36, 55, 64]. The two behavioural ecotypes—migratory (frontal) and resident (coastal)—co–occur along the coasts only during the breeding season, while occupying distinct marine habitats for most of the year [11, 45, 55]. Despite periods of seasonal sympatry, genomic divergence between ecotypes is maintained and is thought to play a key role in ecotypic adaptation, since several genes within these regions are related to differing tolerances to ranges of temperature, salinity, and oxygen availability, perception of dim light, and to brain, muscle, and potentially swim bladder function (Sinclair–Waters et al. [42, 52, 79]. Thus, specific genetic traits are selected in the migratory ecotype for survival in colder waters and movements across greater depth gradients. Conversely, selection of specific traits in the stationary ecotype corresponds to settlement in environments with highly stratified salinity gradients [42, 52]. Furthermore, physiological profiles indicate that the ecotypes exhibit distinct tolerances, with the stationary ecotype likely being better adapted to cope with ocean stressors compared to its migratory counterpart [65]. Exploring ecotype characteristics can therefore provide insight into species adaptation and adaptive potential in a changing environment, particularly for populations inhabiting the North Atlantic and Arctic Oceans, regions exhibiting historically intense overfishing [32] and accelerated thermal dynamics that outpace global trends [24, 35, 73]. In this context, investigating the gut microbiome structure of cod ecotypes can provide insights into the impacts of divergent evolution and of microbiome response to different behaviours and stressors, given that microbial diversity is strongly influenced by lifestyle and health of the host [16]. Moreover, microbiome profiling may provide a complementary tool for stock identification in Atlantic cod populations, including during the spawning season when ecotypes occur in sympatry, integrating this technique into established stock identification frameworks, alongside existing genotype–, otolith–, and morphology–based techniques [39, 42, 95]. Integrating these approaches is essential to better understand how ecotypes respond to external inputs, thereby playing an essential role in possible conservation strategies for several Atlantic cod populations. Current knowledge about the microbiome of cod behavioural ecotypes derives from the stationary Norwegian coastal cod (NCC) and the frontal Northeast Arctic cod (NEAC), the latter having originated from the former and both covering broad marine areas [9, 33]: the coastal ecotype is generally characterised by smaller populations residing along the coasts of Canada, Iceland, Scotland, and Norway, while the frontal ecotype has larger migratory populations, identified in particular off the Grand Bank, on the Newfoundland shelf, in the waters around Iceland and Svalbard, and in the Barents Sea [11, 45]. Previous studies have shown that the gut microbiome of Atlantic cod possesses a microbial structure which may exhibit complex patterns of variation in different populations, depending on extrinsic factors [74, 75]. For instance, the distinction between the gut microbiome of NEAC and NCC was more pronounced than that between cod and other Gadidae species from the same marine area [75], confirming that in teleosts some environmental factors, such as temperature, salinity, and pollution, can be more powerful drivers than host phylogeny [40, 75, 81]. While NEAC and NCC cover such vast areas, other regional ecotypes with smaller populations have been identified in the North Atlantic Ocean: Icelandic cod, for example, likely originated from NEAC but has independently developed frontal and coastal behavioural patterns—although they are genetically less distinct from each other than the NEAC and NCC ecotypes [8, 51, 64]. Building upon previous observations of microbiome divergence between NEAC and NCC, this study investigates for the first time gut microbiome differentiation between the frontal and coastal ecotypes of Icelandic cod, with the aim of assessing whether ecotype divergence is reflected in microbiome divergence at a smaller geographic scale, also considering two different temporal ranges in order to provide insights about the degree of maintenance of the gut microbiome over time. The migratory dynamics as well as biological and genomic characteristics of Icelandic cod have been already studied for decades [37, 51, 63], and yet microbiome research on its behavioural ecotypes is almost absent. Exploring the gut microbiome of Icelandic cod populations represents an important opportunity to: i) further investigate the impact of behavioural ecotypes on the fish microbiome, thereby providing novel insights into the effects of divergent evolution on host–associated microbial communities,ii) improve our understanding of the role of the microbiome in ecological adaptation,and iii) better elucidate how different ecotypes respond to external environmental inputs, ultimately contributing to the refinement of future conservation strategies. Here, we used the metabarcoding approach to characterise the intestinal microbial communities of Icelandic cod, considering coastal and frontal individuals collected in two different timeframes (December 2021 and January 2024) in the North–Iceland marine areas.

## Materials and Methods

### Sampling

**Fig. 1:**
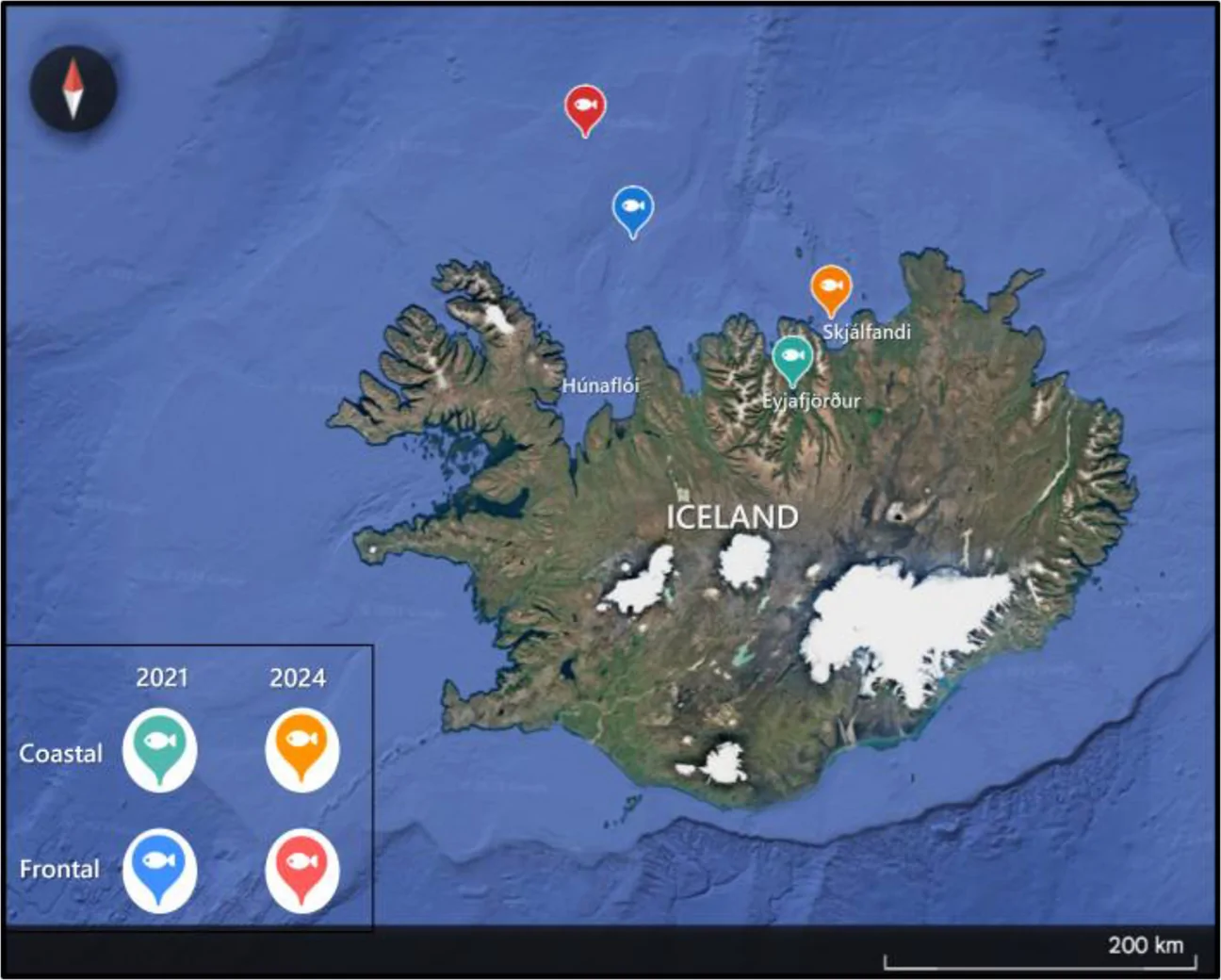
Sampling locations of Atlantic cod (*Gadus morhua*) in Icelandic waters created through Google Earth. In the December 2021 cohort, the coastal group—sampled within Eyjafjörður—is indicated in teal (65°45′00.0"N, 18°07′12.0"W; Depth: 75 m), whereas the frontal group—sampled offshore from Húnaflói—is marked in light blue (66°36′00.0"N, 20°21′00.0"W; Depth: 300 m). In the January 2024 cohort, the coastal group—sampled within Skjálfandi—is indicated in orange (66°08′24.0"N, 17°33′36.0"W; Depth: 75 m), whereas the frontal group—also sampled offshore from Húnaflói—is marked in red (67°10′12.0"N, 21°04′12.0"W; Depth: 304 m). Locations for the frontal and coastal groups correspond to the offshore and fjord clusters, defined via PCA based on sea surface temperatures, depth, and sea bottom temperatures [91]

To characterise the microbial structure of intestinal content in Atlantic cod via 16S rRNA gene amplicon sequencing, 83 samples of fish were collected from the northern marine areas of Iceland. The project spans two sampling periods: December 2021—with 34 individuals in total—and January 2024—with 49 individuals in total. In both periods, coastal and frontal individuals were collected from commercial fishermen (these were not sampled during a research cruise) at periods when the weather cooperated, fishermen were active in the general area of interest, and the University of Iceland researchers were available to process the cod (Fig. 1). Ecotypic designation was determined by capture depth during the feeding season: coastal individuals were collected at depths of 75–86 m, whereas frontal individuals were retrieved at 300–304 m. Both deepwater locations were situated to the north of Húnaflói, whereas coastal locations corresponded to Eyjafjörður (2021 cohort) and Skjálfandi (2024 cohort) (Fig. 1). Based on a Principal Component Analysis of sampling locations offshore and in fjords north of Iceland (2019–2022) (Supplementary Fig. S1), offshore locations north of Húnaflói, including those outlined in Fig. 1, grouped together and separately from those in the fjords, including Eyjafjörður and Skjálfandi (see Supplementary Fig. S2 for reference areas). This clustering was based on of sea surface temperature, depth, and sea bottom temperature [91].

### Samples Processing

Prior to dissection, thawed Atlantic cod individuals were measured for body length and weight by the University of Iceland researchers. Then, fish were dissected and the organs of samples collected in December 2021 were stored at -30 °C, while those of samples collected in January 2024 were stored at -80 °C. Intestinal contents were collected by gentle squeezing under a laminar flow hood, while equipment was sterilised with bleach and alcohol between samples to avoid DNA contamination. Around 250–270 mg of intestinal content for each sample was stored in a screw–cap tube at -30 °C.

### Extraction

DNA was extracted using the Monarch Genomic DNA Purification Kit (New England Biolabs) following the manufacturer's tissue protocol, with specific modifications introduced to optimize total yield. The standard protocol was amended as follows: **i)** immediately after the addition of 10 μL of Proteinase K and 100 μL of Tissue Lysis Buffer, bead beating was performed at 5 m/s for 4 min using 4 steel beads (3 mm in diameter) and 270–300 mg of 0.5–mm diameter glass beads; ii) samples were incubated at 56 °C for 3 h and vortexed for 1 min every 30 min; iii) the centrifugation step prior to adding 3 μL of RNase A was omitted; iv) the RNase A incubation time was extended to 10 min instead of 5 min; v) an additional 3–min dry spin was executed after the second wash step; vi) columns were incubated with 100 μL of Elution Buffer at room temperature for 5 min; and vii) the elution step was repeated twice.

### Sequencing and Bioinformatics

V3–V4 hypervariable region of the 16S rRNA gene was amplified using primers 341F and 785R [41] along with Illumina adaptor overhang sequences and 2 × KAPA HiFi HotStart ReadyMix (KAPA Biosystems). The thermal cycle protocol included an initial denaturation phase at 95 °C for 3 min, 30 cycles of denaturation at 95 °C for 30 s, annealing at 55 °C for 30 s, and extension at 72 °C for 30 s, followed by a final extension step at 72 °C for 5 min. The Illumina protocol "16S Metagenomic Sequencing Library Preparation" was used according to manufacturer’s instructions. Briefly, all PCR amplicons were cleaned up using Agencourt AMPure XP magnetic beads (Beckman Coulter, Brea, CA, USA) and indexed libraries were prepared by limited–cycle PCR using Nextera technology. Indexing was followed by a second clean–up as described above. Libraries were then quantified using a Qubit 3.0 fluorimeter (Invitrogen, Waltham, MA, USA), normalised to 4 nM, and pooled. Prior to sequencing, the sample pool was denatured with 0.2 N NaOH and diluted to 4.5 pM with a 20% PhiX control. Sequencing was performed on an Illumina MiSeq platform using a 2 × 300–bp paired–end protocol, according to the manufacturer’s instructions (Illumina, San Diego, CA, USA). Raw sequences were processed using a pipeline combining PANDAseq [54] and QIIME 2 [12]. High–quality reads (min/max length = 350/550 bp) were retained using the “fastq filter” function of the Usearch11 algorithm [23]. Based on the phred Q score probabilities, reads with an expected error per base E = 0.03 (i.e., three expected errors every 100 bases) were discarded. The retained reads were then binned into amplicon sequence variants (ASVs) using DADA2 [14]. Rarefaction curves were generated for all initial samples to evaluate microbial diversity saturation (Supplementary Fig. S3),based on these curves, samples failing to meet the 905 reads threshold were excluded from subsequent downstream analyses, resulting in the loss of 2 samples. The ASVs were subsequently clustered into Operational Taxonomic Units (OTUs) at a 97% similarity threshold. Taxonomic assignment was performed using the VSEARCH algorithm [76] and the SILVA database [70]. All sequences assigned to eukaryotes (including mitochondrial and chloroplast sequences) or unassigned were discarded. Normalisation was performed to the lowest number of reads for all samples. Beta diversity was estimated by computing weighted and unweighted UniFrac distances. Alpha diversity was calculated by means of three different metrics, namely Faith’s Phylogenetic Diversity, number of observed OTUs and Shannon’s index. The 16S rRNA gene was sequenced from 83 intestinal content samples and following quality control and reads filtering, 81 intestinal samples were retained for downstream analysis.

In order to compare our dataset with NCC and NEAC ecotypes retrieved from literature, we downloaded 30 shotgun sequences of NCC and 30 of NEAC from the dataset of [75]. To make the shotgun sequences comparable with the 16S sequences of our study, samples were processed using PhyloFlash v3.4.2 (Gruber–Vodicka et al. 31 to reconstruct SSU rRNA sequences directly from raw data, a methodology previously applied by Rampelli et al. [71]. Representative SSU sequences from cod samples were aligned with SINA v1.7.2 [69] against the SILVA 138.2 reference alignment. Gap–rich positions (> 95% gaps) were filtered out using TrimAl v1.5.0 (Capella–Gutiérrez et al. 15. Constraint files were subsequently generated, then phylogenetic trees were reconstructed with FastTreeMP v2.1.11–2 [68]. These trees were employed for phylogeny–informed clustering of sequences into OTUs following the approach of Groussin et al. [30].

### Biostatistics Analyses on Atlantic cod Samples

All statistical analyses were conducted in R version 4.4.0 (www.r–project.org) using the following packages: RcppAlgos (v2.9.3; [92], beeswarm (v0.4.0, [26]), made4 (v1.78.0; [17]), cluster (v2.1.6; [50], pairwiseAdonis (v0.4.1, [3], vegan (v2.6–2, [60], tidyverse (v.2.0.0, [88], matrixStats(v.1.5.0, [7]), dplyr (v.1.1.4; [90], stringr (v.1.5.1, [87], xlsx (v0.6.5, [22].

To compare Icelandic cod, NEAC and NCC, the Bray–Curtis distance was calculated to compare the datasets at the genus level, then MDS1 was plotted using the beeswarm package. The genus level was selected in this analysis to provide the highest possible phylogenetic resolution for the comparison between NEAC, NCC, and our dataset, while excluding unassigned diversity. For all the other comparison analyses, OTUs were used.

Both alpha and beta diversity were measured using, respectively, Faith’s Phylogenetic Diversity (Faith_PD), Observed Features, and Shannon Entropy for alpha diversity, and UniFrac distances (both weighted and unweighted) for beta diversity. UniFrac distance matrices were subsequently used as input for Principal Coordinates Analysis (PCoA) to visualize compositional dissimilarities among samples. Alpha diversity statistical differences between the two groups discriminated by ecotype and the groups discriminated by year were evaluated using the Wilcoxon rank–sum test, while beta diversity statistical differences were evaluated using Permutational Multivariate Analysis of Variance (PERMANOVA) based on pseudo–F ratios via the adonis2 function of the vegan R package (v. 2.6–2). To support the interpretation of beta diversity patterns, the homogeneity of multivariate dispersions was assessed using the betadisper function in the vegan R package (v. 2.6–2). The statistical significance of the differences in dispersion was evaluated using Analysis of Variance (ANOVA). To evaluate environmental influences on beta diversity across sampling periods, sample coordinates along the MDS1 axis were extracted from PCoA shown in Fig. 6c–d–e–f. Relationships between these ordination scores and 11 environmental parameters (see paragraph *Data acquisition and statistics of environmental parameters on the North Icelandic sampling area*) were then assessed using non–parametric Spearman’s rank correlations. To quantify yearly intergroup differences in beta diversity, we computed the mean weighted UniFrac distance of each sample to all samples belonging to the opposite ecotype group (Coastal vs. Frontal) using a direct measure of ecological separation between ecotypes, visualized through boxplots and statistically tested using PERMANOVA with 999 permutations, based on *pseudo*–*F ratios* via the adonis2 test of the vegan R package (v. 2.6–2). Linear Discriminant Analysis Effect Size (LEfSe) was then applied to identify taxa discriminating among years and ecotypes [77], with statistical significance set at p < 0.05 and LDA score cutoff set at 1.5,the taxonomy was manually assigned (NCBI BLAST). Lastly, a bipartite network analysis was performed using Cytoscape (v3.10.3; [78] to visually represent the distribution of OTUs across the samples, using the OTU tables obtained from the upstream bioinformatic pipeline and without applying any additional abundance or prevalence filtering prior to network construction. Within the network, samples are represented as rhombuses and individual OTUs as dots. Shared taxa were defined as OTUs detected in at least one sample across all analysed groups. Conversely, exclusive taxa were defined as OTUs present in at least one sample of a specific group but completely absent in all samples of the alternative group.

### Data Acquisition and Statistics of Environmental Parameters on the North Icelandic Sampling Area

Data values of environmental parameters were retrieved from the E.U. Copernicus Marine Service Information (CMEMS) website (https://marine.copernicus.eu/it), part of the European Union's Earth Observation Program, using the datasets GLOBAL_MULTIYEAR_PHY_001_030, Accessed 14–11–2025, https://doi.org/10.48670/moi-00021, GLOBAL_MULTIYEAR_BGC_001_029, Accessed 14–11–2025, https://doi.org/10.48670/moi-00019. The marine area (Supplementary Fig. S4) was chosen according to the knowledge about areas where coastal and frontal cod ecotypes in North Iceland waters are usually found according to Pampoulie et al. [63]. Environmental parameters considered were sea water potential temperature (degrees C), pH reported on total scale (1), chlorophyll–a (mg/m^3^), dissolved iron (mmol/m^3^), dissolved molecular oxygen (mmol/m^3^), carbon dioxide (Pa), nitrate (mmol/m^3^), phosphate (mmol/m^3^), silicate (mmol/m^3^), phytoplankton (mmol/m^3^), net primary production of biomass (mg/m^3^*day). All statistical analyses were conducted in R version 4.4.0 (www.r–project.org) using the following packages: tidyverse (v2.0.0, [88], readxl (v1.4.5, [89], ggplot2 (v4.0.2, [86]), PMCMRplus (v1.9.12; [67], and multcompView (v0.1–10, [29]. The linegraphs were created using Excel (Microsoft 365). The boxplot and heatmap representations showing colour gradients according to the values get from the CMEMS website were generated using ggplot function in R, reporting the years from 2019 to 2024 in rows and the months from January to December in columns. Statistical differences between annual distribution of values were evaluated using the Kruskal–Wallis test. As further investigation, statistical analyses of environmental parameters (2019–2023) are reported and described in the Supplementary Information–Analyses on environmental parameters retrieved by E.U. Copernicus Marine Service Information (CMEMS).

It is important to note that the publicly available datasets employed in this study were designed to capture large–scale environmental patterns and shallow–water conditions rather than to provide a precise characterisation of the localized physicochemical environments experienced by individual cod populations. Accordingly, the abiotic variables considered here should be interpreted as macro–scale environmental descriptors, used to investigate whether broad environmental gradients may contribute to the observed variation in gut microbiome composition.

## Results

### Characterisation of Icelandic cod Gut Microbiome and Differentiation from NEAC and NCC

Icelandic cod individuals were firstly morphologically characterised considering body length and weight (Supplementary Tab. S1). Consistent with known ecotypic traits, frontal cod displayed greater mean lengths and weights, alongside more pronounced inter–individual variability (Supplementary Tab. S2). To characterise the microbial community profiles associated with these ecotypes, we performed metabarcoding analyses on intestinal contents from 83 Icelandic cod. After the filtering process, 81 samples were retained for downstream analyses: 12 coastal and 21 frontal cod collected in December 2021, and 27 coastal and 21 frontal cod collected in January 2024. Samples had a mean sequencing depth of approximately 80,000 reads after quality filtering (± 37,000 SD).

**Fig. 2:**
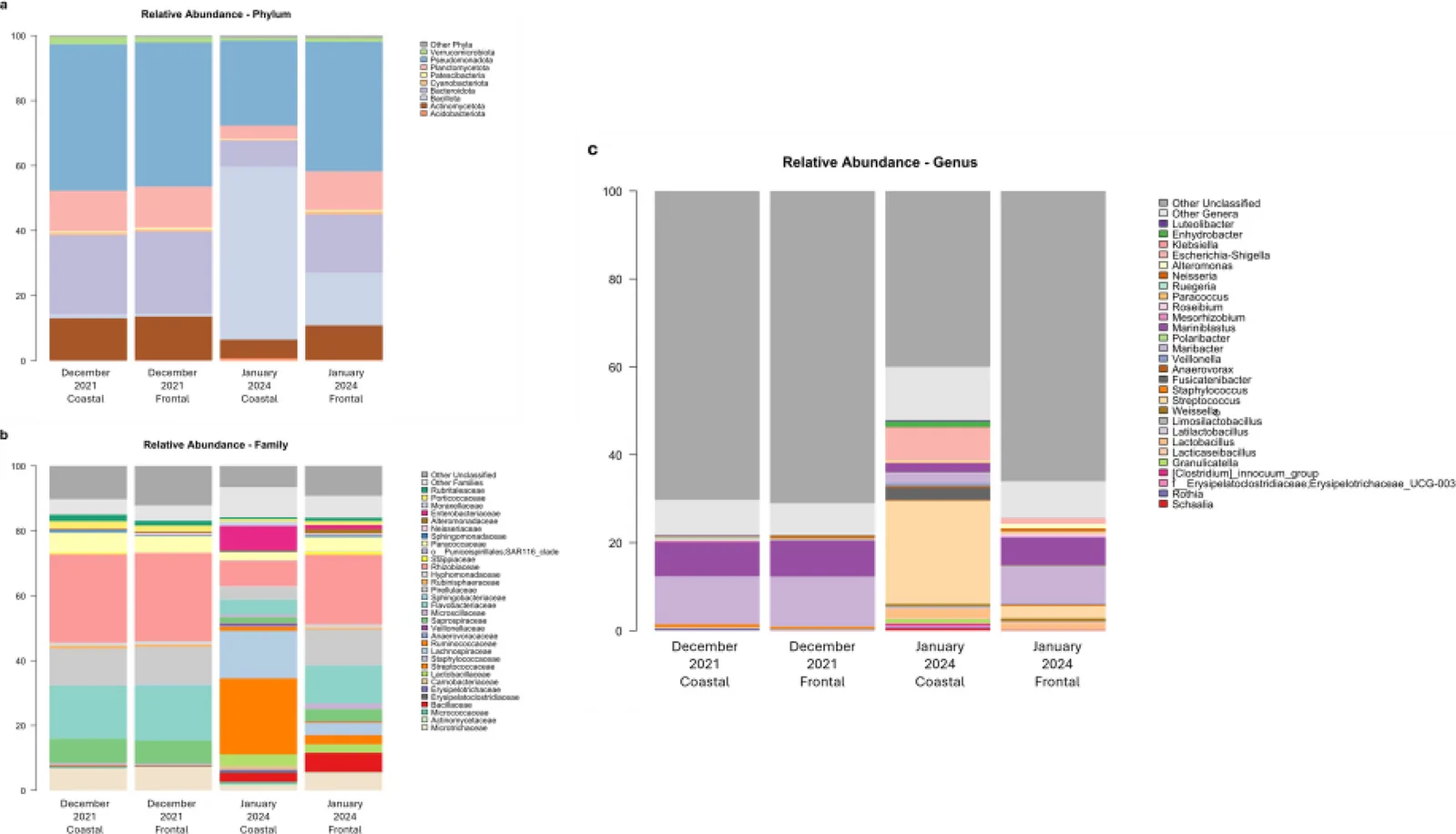
Taxonomic composition of the Atlantic cod gut microbiome. Barplots summarizing bacterial composition at **a** phylum, **b** family and **c** genus levels of both ecotypes for each timeframe. Microbial phyla, families and genera with relative abundance < 2% were filtered as “others”

Gut microbial composition was characterised at the phylum, family, and genus levels. A significant degree of individual specificity was observed in the Icelandic cod gut microbiome. In terms of general taxonomic composition, the dominant phyla in the Icelandic cod gut microbiome were *Pseudomonadota* and *Bacteroidota* (Fig. 2a) showing the highest mean relative abundance, respectively, of 45.0% ± 8.3% and 25.0% ± 5.0%, while *Actinomycetota* and *Planctomycetota* constituted subdominant phyla (respectively, 13.5% ± 4.9% and 12.6% ± 4.2%). At the family level (Fig. 2b), the most represented taxa were *Rhizobiaceae* (27.2% ± 7.2%), *Flavobacteriaceae* (16.9% ± 3.5%), *Pirellulaceae* (11.6% ± 3.9%), accompanied by a substantial proportion of unclassified families (12.2% ± 5.0%). At lower taxonomic ranks (Fig. 2c), most taxa remained unclassified (71.0% ± 5.7%); among identified genera, *Maribacter* showed the highest abundance (11.3% ± 2.9%).

**Fig. 3:**
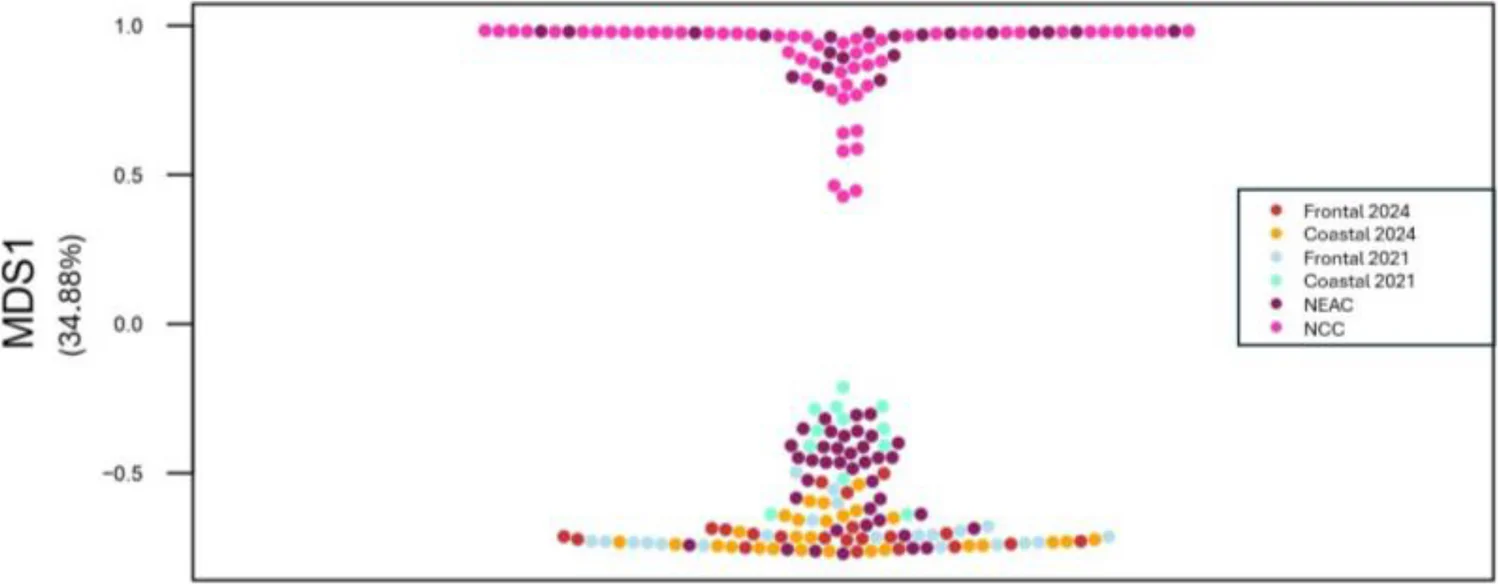
Beeswarm plot representing the Bray–Curtis distances at the genus level between different ecotypes of Icelandic cod (our dataset) as well as NEAC and NCC of the Lofoten area retrieved from literature [75]. MDS1 (34.88%) was plotted on the y–axis

To provide an overview on the gut microbiome differentiation between frontal and coastal ecotypes of Icelandic cod within the broader context of the migratory NEAC and the resident NCC, we performed a comparative analysis of the Icelandic samples collected in this study and previously published NEAC and NCC samples collected in the Lofoten area by Riiser et al. [75]. The beeswarm plot of the first MDS axis explaining 34.88% variance of Bray–Curtis distance between gut microbiome samples (Fig. 3) revealed two clusters of samples: one cluster included all NCC individuals along with few NEAC individuals, while the other included all Icelandic cod individuals (frontal and coastal ecotypes of December 2021 and January 2024) and the majority of NEAC individuals.

### Variation of the cod Gut Microbiome between Coastal and Frontal Ecotypes Collected in December 2021 and January 2024

To assess differences in microbial composition between coastal and frontal ecotypes, both alpha and beta diversity were analysed for December 2021 cohort (a–c–e) and for January 2024 cohort (b–d–f).

**Fig. 4:**
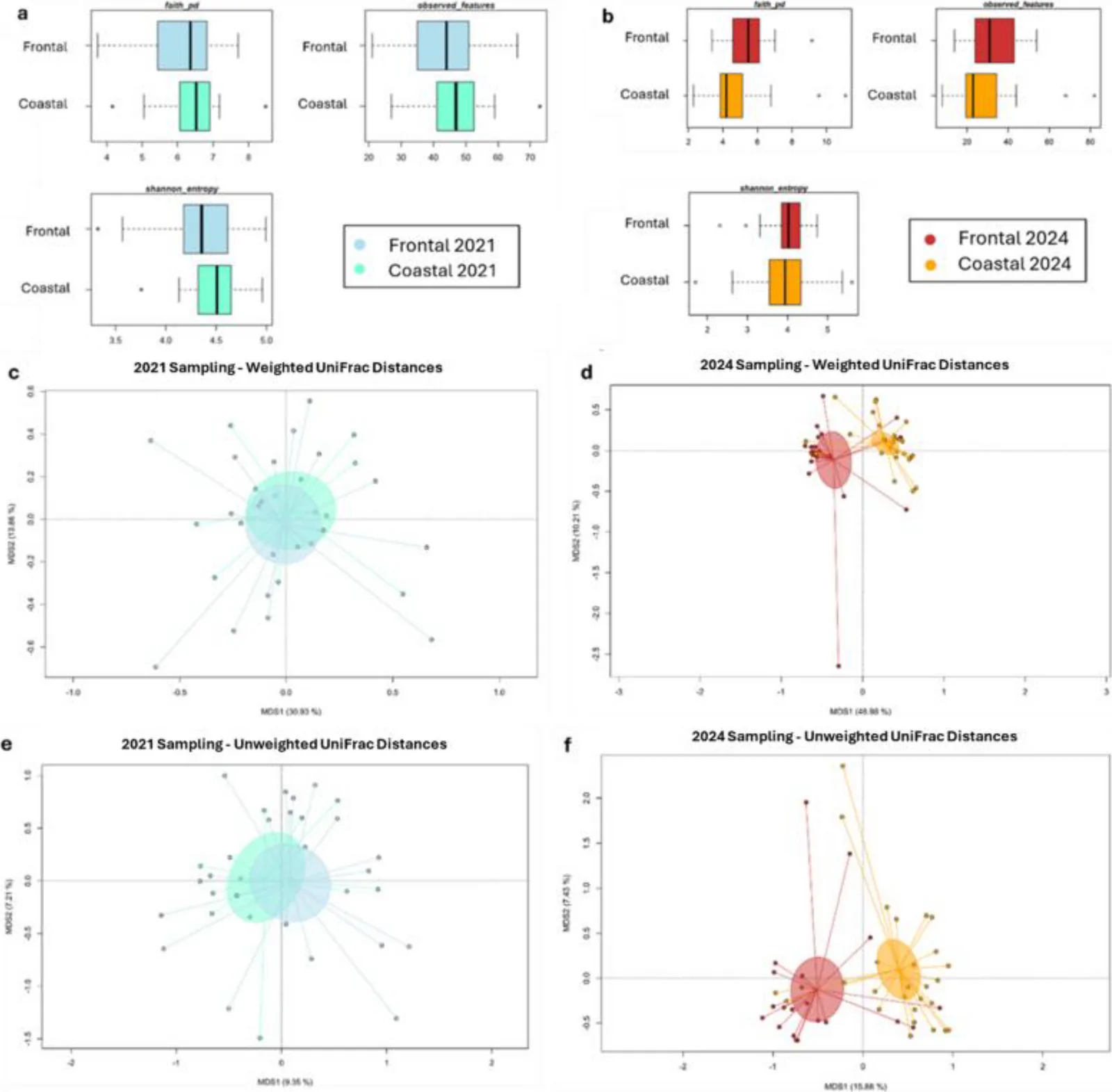
Alpha and beta diversity of gut microbial community variation between coastal (teal) and frontal (light blue) cod collected in December 2021 (a–c–e) and between coastal (orange) and frontal (red) cod collected in January 2024 (b–d–f). **a**–**b** Boxplots show microbial alpha diversity values in December 2021 cohort (a) and in January 2024 cohort (b) individuals measured by Faith’s Phylogenetic Diversity (faith_pd), observed features and Shannon entropy. All metrics displayed no significant variation (Wilcoxon rank–sum test *p* > 0.05) of alpha diversity between ecotypes groups in the gut. **c–d–e–f** Beta diversity of gut of cod collected in December 2021 (c–e) and those collected in January 2024 (d–f). PCoA based on weighted and unweighted UniFrac distances between ecotypes. Cod samples collected in December 2021 do not show a significant separation between ecotypes, whereas those collected in January 2024 are significantly separated (permutation test with PERMANOVA using the adonis2 function; *p* < 0.001). Multivariate homogeneity of groups dispersions tested via betadisper was not significant for all comparisons (ANOVA *p* > 0.05)

Considering the December 2021 cohort, the alpha diversity (within–group diversity) was measured using three different metrics (Faith’s PD, observed features, and Shannon entropy) (Fig. 4a) and, according to the Wilcoxon rank–sum test, no significant differences were found. Furthermore, beta diversity (between–group diversity) was assessed via Principal Coordinate Analysis (PCoA) based on weighted and unweighted UniFrac distance metrics, which revealed no significant separation in the microbial community structure (Fig. 4c–e). The assessment of multivariate homogeneity of groups dispersions (betadisper function) followed by ANOVA revealed no significant differences in dispersion across ecotypes (*p* > 0.05). To ensure methodological consistency, the January 2024 cohort was subjected to the same diversity analyses used for the 2021 samples to investigate the ecotype–associated variation. While alpha diversity showed no significant differences across any of the three metrics (Fig. 4b), beta diversity analysis revealed significant segregation (Fig. 4d–f). Finally, the January 2024 cohort showed no significant differences in dispersion across ecotypes (*p* > 0.05), confirming that the variance within each group was homogenous for both sampling timepoints.

**Fig. 5:**
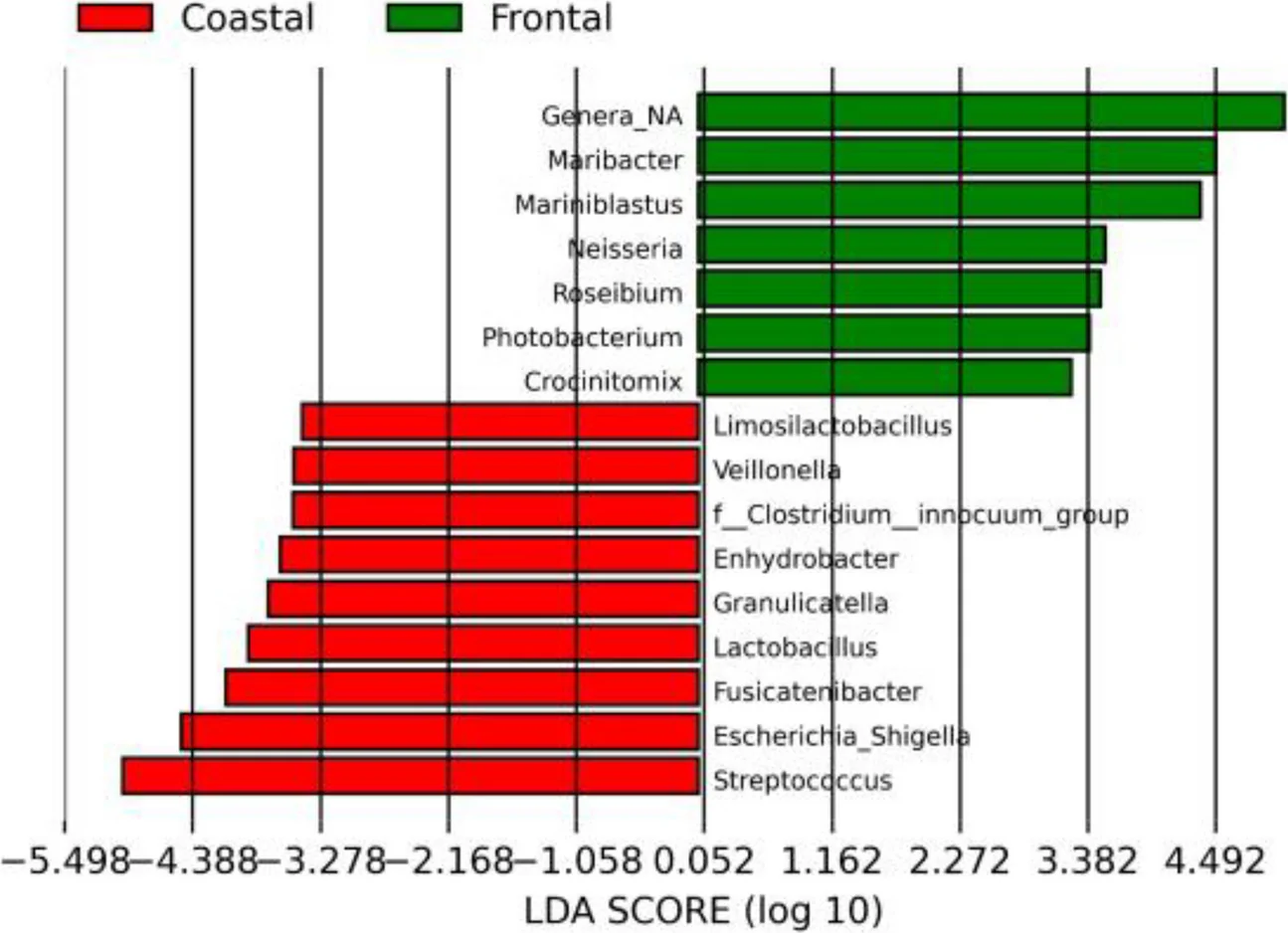
LEfSe analysis performed on gut composition of coastal and frontal ecotypes for the year 2024 with LDA score cutoff of 1.5 and a *p* < 0.05

To further investigate microbial differentiation, both discriminant and shared taxa among frontal and coastal cod groups were determined at the genus level. For the discriminant taxa characterisation, the Linear Discriminant Analysis Effect Size, or LEfSe, with LDA score cutoff of 1.5 and *p* < 0.05 was performed, separately, for each year. For cod individuals collected in December 2021, LEfSe analysis of the gut samples identified two discriminant taxa for the coastal ecotype: *Marinomonas* and OM27 clade (Supplementary Fig. S5a). Based on rarefied OTU counts, a total of 121 out of 789 OTUs (15.3%) were shared between coastal and frontal ecotypes, with a mean abundance of 6.5 reads per OTU (Supplementary Fig. S5b). Despite representing a minor fraction of the total detected nodes, this shared subset potentially accounted for a major fraction of the cumulative relative abundance and phylogenetic diversity within the microbial communities. Conversely, the large proportion of ecotype–specific OTUs might consist primarily of rare, low–abundance components. This potential dominance of a shared core microbiome could explain the limited segregation observed in the beta diversity ordination analyses, despite the high number of non–shared rare taxa. In the January 2024 cohort, LEfSe analysis identified six discriminant genera for the frontal ecotype—*Maribacter*, *Mariniblastus*, *Neisseria*, *Roseibium*, *Photobacterium*, *Crocinitomix*—and nine for the coastal ecotype—*Limosilactobacillus*, *Veillonella*, *Clostridium innocuum group*, *Enhydrobacter*, *Granulicatella*, *Lactobacillus*, *Fusicatenibacter*, *Escherichia*–*Shigella*, *Streptococcus* (Fig. 5). Finally, the analysis of OTUs sharing was performed on the January 2024 cohort and represented as OTUs–sharing network (Supplementary Fig. S6). The analyses revealed that 83 out of 917 OTUs (9%) were shared between coastal and frontal ecotypes, with a mean abundance of 7.5 reads per OTU.

### Exploring the Gut Microbiome Variation of Icelandic cod According to the Sampling Periods

**Fig. 6:**
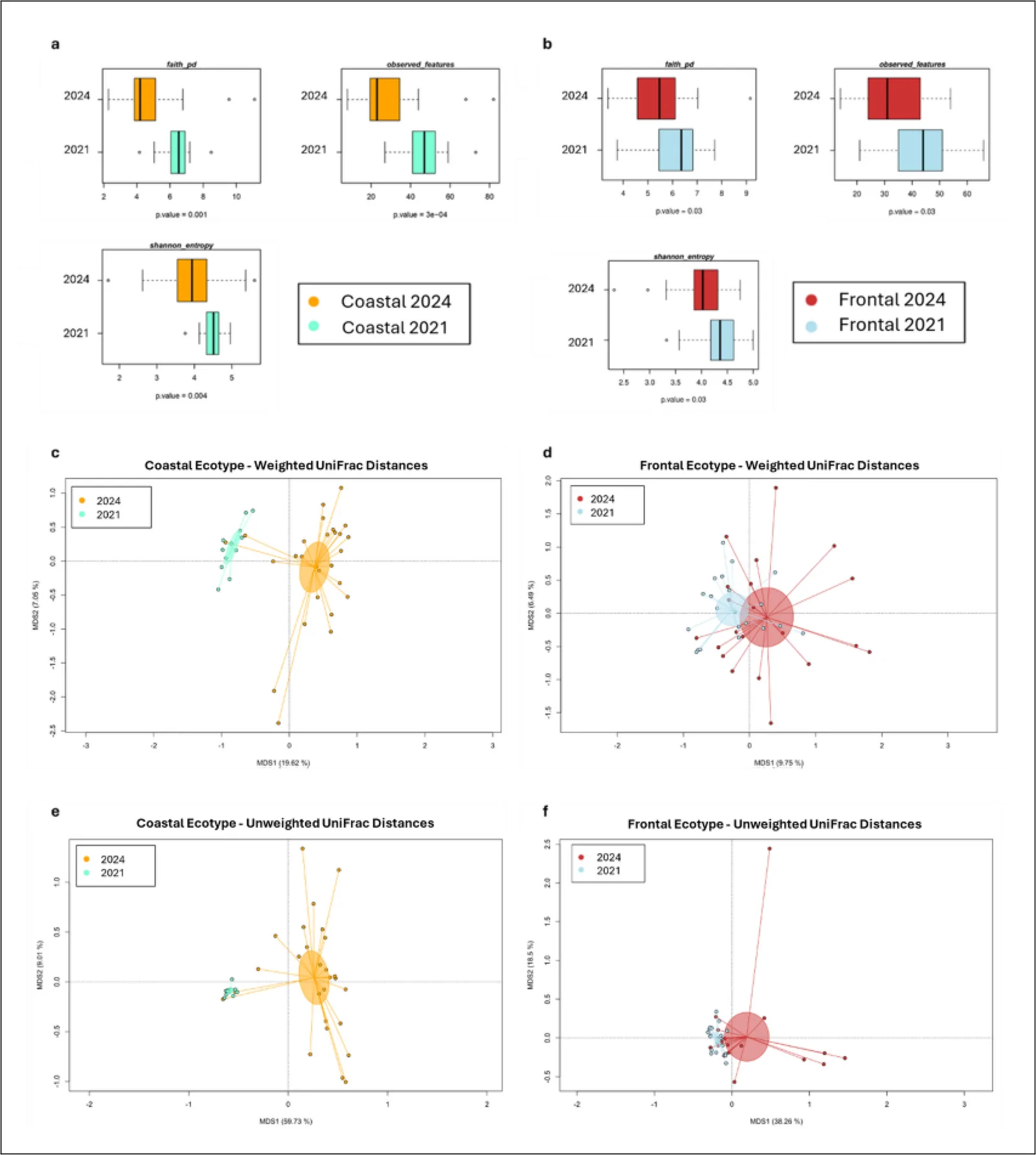
Alpha and beta diversity of gut microbial community variation between coastal cod (a–c–e) collected in December 2021 (teal) and those collected in January 2024 (orange), and between frontal cod (b–d–f) collected in December 2021 (light blue) and those collected in January 2024 (red). **a–b** Boxplots show alpha diversity values measured by Faith’s Phylogenetic Diversity (faith_pd), observed features and Shannon entropy for coastal and frontal ecotype. All metrics displayed significant variation (Wilcoxon rank–sum test *p* < 0.05) of alpha diversity along years. **c–d–e–f** Beta diversity of gut of coastal and frontal ecotype. PCoA based on weighted and unweighted UniFrac distances between years. Samples are significantly separated (permutation test with PERMANOVA using the adonis2 function; Coastal: *p* < 0.001; Frontal: *p* < 0.05). Multivariate homogeneity of groups dispersions tested via betadisper was significant for all comparisons (ANOVA < 0.05). Spearman’s rank correlation analysis between sample coordinates along the MDS1 axis and 11 environmental variables showed significant relationships for the coastal ecotype (*p* < 0.001 for both weighted and unweighted UniFrac), whereas no significant correlations were detected for the frontal ecotype (*p* > 0.05). Detailed correlation coefficients and p–values are provided in Supplementary Table S3

Because ecotype–associated microbiome differentiation differed between sampling periods, a further analysis was carried out looking at the variation of the same ecotype between the sampling periods. We investigated the annual gut microbiome changes for frontal and coastal ecotypes comparing, firstly, the Icelandic frontal cod collected in December 2021 with those collected in January 2024 and then comparing Icelandic coastal cod collected in December 2021 with those collected in January 2024. The gut microbial composition between cod collected in December 2021 and those in January 2024 were analysed through the alpha and beta diversities, comparing, separately, all coastal cod (December 2021 vs January 2024) and all frontal cod (December 2021 vs January 2024) individuals. Alpha diversity values were measured by Faith’s Phylogenetic Diversity (faith_pd), observed features, and Shannon entropy metrics. Then a Wilcoxon rank–sum test was performed to assess the significance of the differences. For coastal and frontal ecotypes, alpha diversity displayed significant variation between December 2021 and January 2024 with all the metrics (*p* < 0.05) (Fig. 6a–b), with January 2024 cohorts being generally characterised by a decrease in the gut microbiome diversity. Further, the PCoA based on weighted (Fig. 6c–d) and unweighted (Fig. 6e–f) UniFrac distances of gut content microbiota was performed for the coastal and frontal ecotypes. Statistical significance of beta diversity differences was assessed using permutation tests with PERMANOVA via the adonis2 function, revealing significant temporal variation across years in both frontal (*p* < 0.05) and, more markedly, coastal (*p* < 0.001) ecotypes.

The homogeneity of multivariate dispersions were evaluated separately for each ecotype. For the coastal ecotype, the betadisper analysis followed by ANOVA revealed a significant difference (*p* < 0.05) in community dispersion between the two sampling events, considering both unweighted (*p* < 0.05) and weighted distances (*p* < 0.001). Although PERMANOVA reveale significant differences in community structure between groups, beta–dispersion analysis was also significant, indicating unequal within–group variability. In particular, samples collected in 2024 showed higher interindividual variability in microbiome composition, resulting in greater dispersion in the ordination space and suggesting a possible dysbiotic transition consistent with the Anna Karenina principle [94]. A similarly significant shift in multivariate dispersion across timepoints was observed for the frontal ecotype (PERMANOVA, *p* < 0.05).

To explore the relationship between microbial community structure and environmental conditions, Spearman rank correlations were performed between the ordination scores of the MDS1 axis, derived from weighted and unweighted UniFrac distance matrices, and 11 environmental variables on the North Icelandic sampling area from 2019 to 2024. Data values of environmental parameters were retrieved from the E.U. Copernicus Marine Service Information (CMEMS) website (see Materials and Methods section for details) and included: sea water potential temperature (°C), pH reported on total scale (1), chlorophyll–a (mg/m^3^), dissolved iron (mmol/m^3^), dissolved molecular oxygen (mmol/m^3^), carbon dioxide (Pa), nitrate (mmol/m^3^), phosphate (mmol/m^3^), silicate (mmol/m^3^), phytoplankton (mmol/m^3^), and net primary production of biomass (mg/m^3^*day). Analyses were conducted separately for the coastal and frontal ecotypes considering, for each variable, the average value corresponding to the month of sampling for each cohort (December 2021 and January 2024). Correlation coefficients and associated p–values are reported in Supplementary Table S3. Significant correlations between the 11 environmental variables and the MDS1 axis were detected for the coastal ecotype under both weighted and unweighted UniFrac metrics (*p* < 0.001), whereas no significant associations were observed for the frontal ecotype (*p* > 0.05), suggesting the absence of relevant associations between environmental drivers and microbial community variation in this group.

**Fig. 7:**
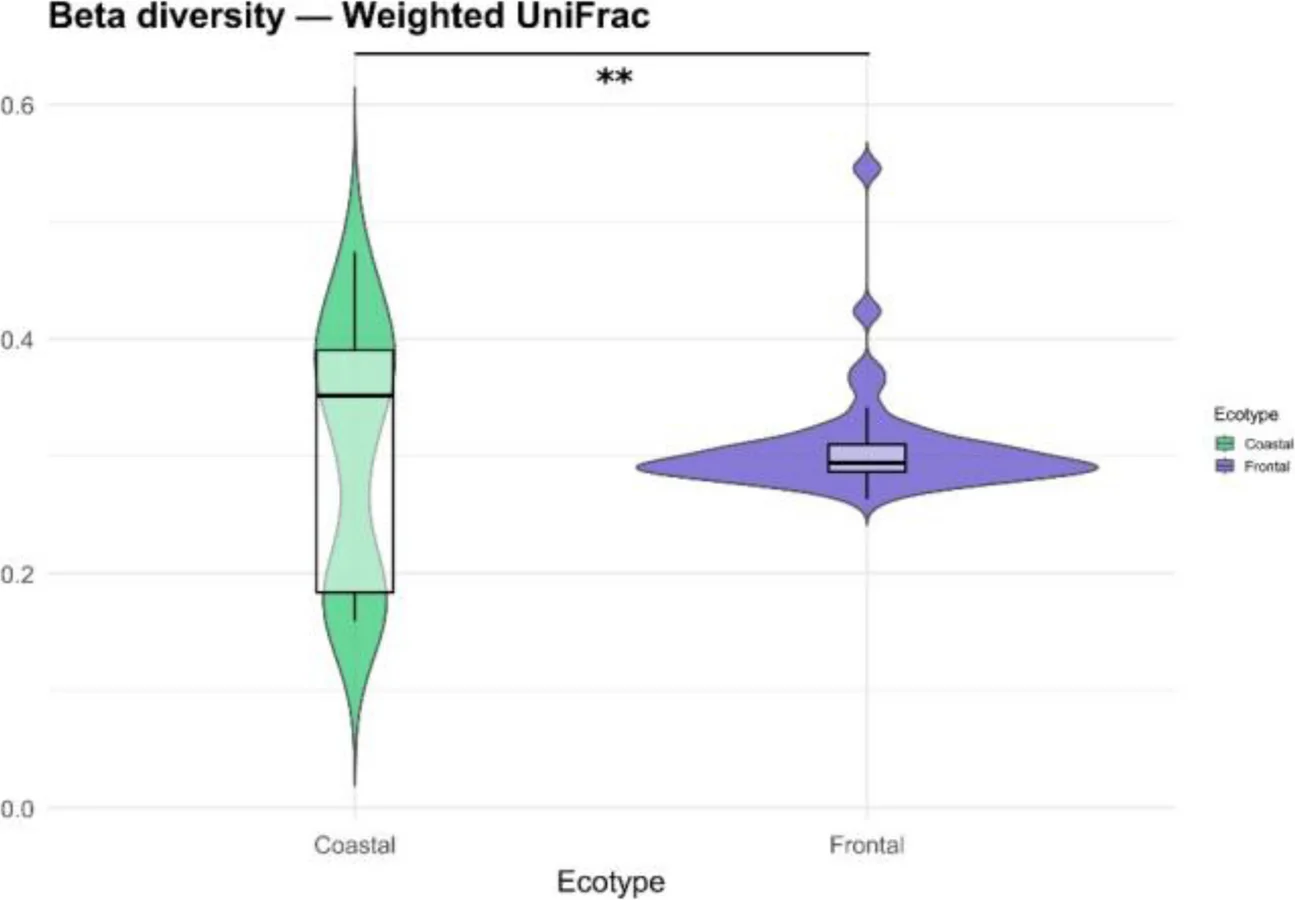
Beta diversity comparison between coastal and frontal ecotypes grouped regardless of the year (December 2021 and January 2024) and based on weighted UniFrac distances. The violin plot shows the distribution of mean weighted UniFrac distances between gut microbial communities from all coastal cod and all frontal cod individuals collected. Groups are significantly different (PERMANOVA using the adonis2 function; *p* < 0.01)

To compare the extent of variation in the two sampling periods between coastal and frontal ecotypes, we assessed gut microbiome changes between December 2021 and January 2024 sampling campaigns by quantifying the corresponding annual differences in weighted UniFrac distances. Figure 7 presents a violin plot showing a significantly higher variation between the two sampling periods in gut microbiome structure in the coastal ecotype compared to the frontal ecotype (PERMANOVA using the adonis2 function; *p* < 0.01).

**Fig. 8:**
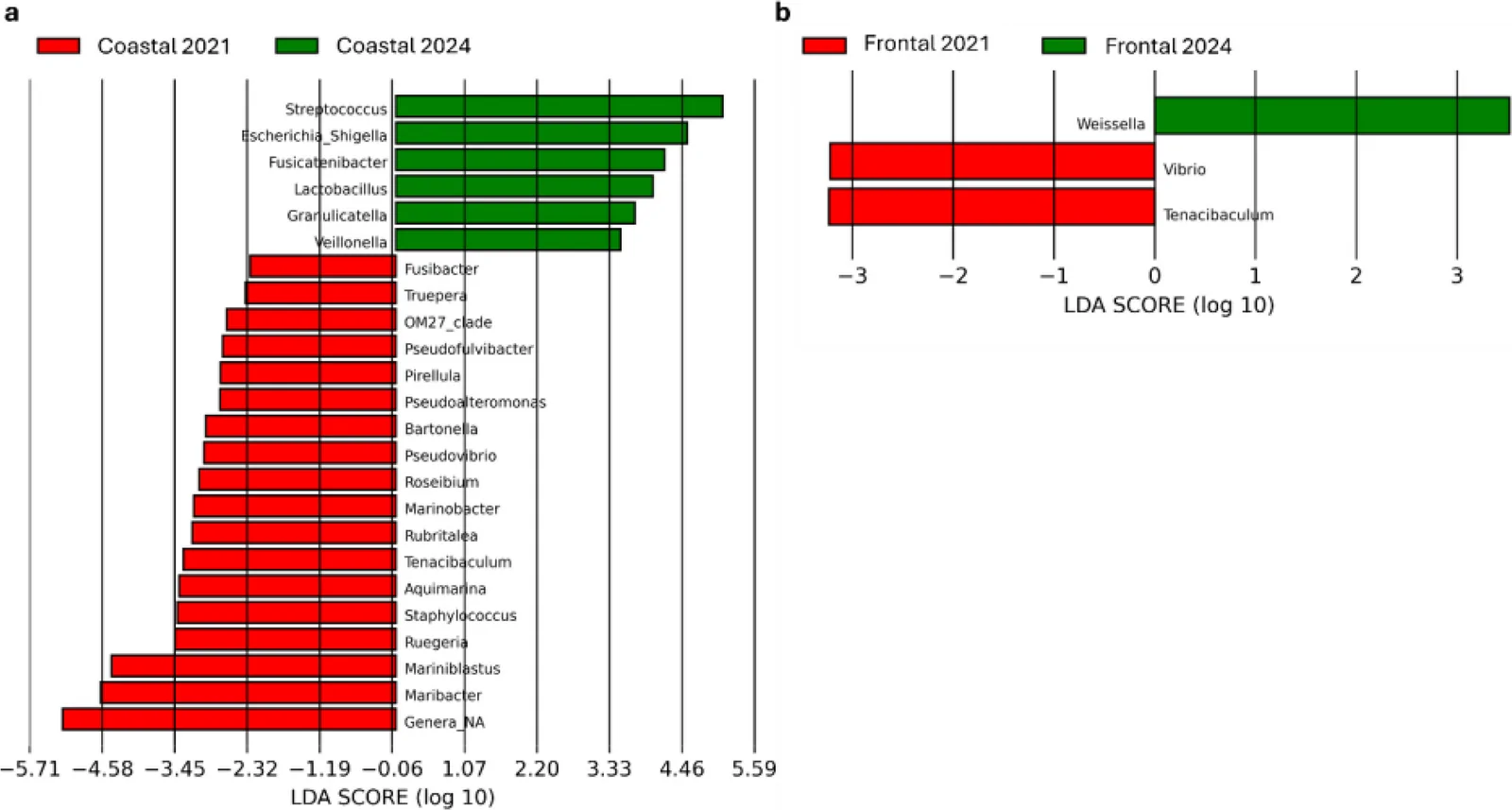
LEfSe analysis performed on gut composition of coastal and frontal ecotypes compared by years with LDA score cutoff of 1.5 and a *p* < 0.05. **a** LEfSe analysis showing discriminant genera of the coastal ecotypes in December 2021 and January 2024 cohorts. **b** LEfSe analysis showing discriminant taxa of the frontal ecotypes in December 2021 and January 2024 cohorts

To investigate the discriminating microbial taxa within cod individuals of the same ecotype according to the sampling period, the LEfSe analyses (LDA score cutoff of 1.5 and *p* < 0.05) were first performed considering coastal cod collected in December 2021 and in January 2024, then considering frontal cod collected in December 2021 and in January 2024. For the coastal ecotype (Fig. 8a), *Fusibacter*, *Truepera*, OM27 clade, *Pseudofulvibacter*, *Pirellula*, *Pseudoalteromonas*, *Bartonella*, *Pseudovibrio*, *Roseibium*, *Marinobacter*, *Rubitalea*, *Tenacibaculum*, *Aquimarina*, *Staphylococcus*, *Ruegeria*, *Mariniblastus*, and *Maribacter* were found as significant genera in December 2021 and *Streptococcus*, *Escherichia*–*Shigella*, *Fusicatenibacter*, *Lactobacillus*, *Granulicatella*, and *Veillonella* in January 2024. For the frontal ecotype (Fig. 8b), *Weissella* was identified as a discriminant genus in January 2024 and *Vibrio* and *Tenacibaculum* in December 2021.

### Survey of Environmental Parameters on the North Icelandic Sampling Area from 2019 to 2024

Looking at the inconsistent trends of the gut microbiome diversification between ecotypes for the sampling carried out in December 2021 and the one carried out in January 2024, we investigated the variability of environmental parameters in the two sampling periods. In this perspective, taking into account the marine area features where coastal and frontal cod ecotypes in North Icelandic waters have been collected for this study and are usually found [63], we looked at the variation between the two sampling periods of important physical and biochemical parameters considering their monthly mean in the period prior from January 2019 to January 2024. Since the 2024 sampling was carried out in January, the trend for 2023 will be used as the environmental reference. Analyses were carried out considering the following environmental parameters: surface potential temperature, pH, net primary production of biomass, carbon dioxide surface pressure, and concentration of dissolved molecular oxygen, chlorophyll–a, phytoplankton, dissolved iron, phosphate, silicate, and nitrate. Annual and monthly trends of each environmental parameter are reported as linegraphs (Supplementary Fig. S7), as heatmaps (Supplementary Fig. S8, left side) and as boxplots showing annual median values (Supplementary Fig. S8, right side). Considering annual trends and median values along the sampling years, 2020 and December 2021 were characterised by lower seawater temperatures and higher oxygenation, along with slightly depleted nutrient levels (phosphates, silicates, and nitrates). In contrast, 2023 and 2024 exhibited an overall opposite pattern, with monthly higher water temperatures, increased CO₂ and nutrient concentrations, as well as lower pH and dissolved oxygen, indicating more eutrophic conditions. While most parameters do not follow a chronological trend over the years, results show how carbon dioxide has risen year after year and, conversely, pH has decreased year after year, reaching their respective maximum and minimum values in 2023. Despite these trends, the Kruskal–Wallis test did not show any significant differences in environmental parameters between these years. Further statistical analyses exploring environmental changes over the period 2019–2023 in the sampling area of this study are provided in the Supplementary Information–Analyses on environmental parameters retrieved by E.U. Copernicus Marine Service Information (CMEMS).

## Discussion

This study presents the first characterisation of the gut microbiome of Icelandic cod, focusing on differences in gut microbial community composition between frontal and coastal ecotypes across two sampling periods, December 2021 and January 2024. To precisely contextualize the temporal comparison, the specific timeline of the sampling design should be noted. Samples were collected within targeted windows, specifically between December 6–9, 2021, and January 16–25, 2024. Given the slight shift in sampling months, the comparison between December 2021 and January 2024 should be interpreted as a meaningful snapshot of potential interannual variation, while acknowledging the minor fluctuations that may naturally occur within the winter season. Consequently, these temporal patterns are conservatively discussed here as period–specific profiles, which may capture broad temporal variations, avoiding any overinterpretation as strictly year–to–year dynamics. Temporal variability is also reflected in cod sizes. Although not aged, based on mean weights, cod sampled in 2021 were heavier, while those sampled in 2024 were lighter, than the long–term average, respectively [53].

Our results indicate that both Icelandic cod ecotypes share gut microbiome similarities with the Northeast Arctic cod (NEAC), the ecotype from which they originated. While environmental conditions and dietary habits generally exert a stronger influence on microbial community composition than host genetics, phylosymbiotic patterns have been detected in marine teleosts [34, 56]. These evolutionary signals may, at least in part, account for the residual microbiome similarities observed between the Icelandic cod ecotypes and NEAC. Although these findings are consistent with the closest relationship between the NEAC and Icelandic cod, this comparison should be interpreted as an exploratory ecological contextualisation rather than a direct quantitative integration of the datasets, because of methodological differences between the sequencing approaches used in the two studies. Interestingly, a deeper comparison of the gut microbiome between Icelandic cod ecotypes was carried out through alpha and beta diversity analyses, revealing contrasting outcomes between the December 2021 and January 2024 cohorts.

In December 2021, coastal and frontal ecotypes did not exhibit substantial differences in microbiome composition, with only a few bacterial taxa discriminating between ecotypes. In contrast, the January 2024 cohort showed pronounced divergence in gut microbiome composition, with a substantially higher number of ecotype–specific bacterial taxa. Our results suggest that this pattern is primarily attributable to substantial variation in the gut microbiome of the coastal ecotype between December 2021 and January 2024, although a detectable variation was also observed in the frontal ecotype across the two sampling periods. Confirming this, we observed a substantial overlap between the gut microbiome taxa characterising the coastal ecotype in January 2024 relative to December 2021 and those distinguishing the coastal ecotype from the frontal one in January 2024.

Investigating the specifics of the observed compositional changes, in December 2021 only two taxa differed significantly in abundance between the coastal and frontal ecotypes, namely *Marinomonas* and the OM27 clade, both discriminating the coastal group from the frontal one and being already detected in marine environments [93], Al–Ansari 1. By contrast, in January 2024 the gut microbiome of Icelandic cod showed pronounced ecotype–specific differentiation, with 16 taxa discriminating between frontal and coastal individuals. In the frontal group nearly all characterising taxa are commonly found in marine environments. Among them, *Photobacterium* is one of the most common genera in fish gut as chitinase–producing gut bacteria [80]. *Maribacter*, *Mariniblastus*, and *Roseibium* have been reported as free–living or holobiont–associated (*e.g.* macroalgae) marine taxa [44, 46, 96], while *Crocinitomix* is a cold–adapted genus detected in both environmental marine samples and Atlantic macroalgae [13]. As for the coastal group, some of the characterising taxa are common inhabitants of the marine fish gut microbiome, such as *Limosilactobacillus*, *Lactobacillus*, *Enhydrobacter*, and *Granulicatella*, whereas others are host–associated bacteria known to be part of the mammalian gut microbiome, including *Clostridium*, *Fusicatenibacter*, *Streptococcus*, and *Escherichia–Shigella*. More specifically, *Limosilactobacillus* and *Lactobacillus* are common harmless commensal bacteria of fish gut which are often used as probiotic in aquaculture enhancing immunity in different water organisms [47, 57]. *Enhydrobacter* is commonly found in the skin mucus of fish but is also known to play a role in the gut as a cellulose–degrading bacterium [10, 82]. In contrast, *Clostridium* and *Fusicatenibacter* are typically detected in the mammalian gastrointestinal tract [18]. Finally, the genera *Streptococcus* and *Escherichia–Shigella* represent notable host–associated taxa, including several opportunistic pathogens affecting both humans and fish, whose dissemination is frequently driven by anthropogenic activities [19, 59, 83]. Although LEfSe analyses may be affected by false–positive discoveries, the identified taxa should be conservatively interpreted as exploratory microbial signatures [58]. Despite these methodological cautions, LEfSe remains a robust and widely recognized tool for initial biomarker discovery in microbiome research, used in this case for unraveling the complex influence of ecotypes on the fish gut microbiome.

Interestingly, the analysis of environmental parameters in the northern Iceland sampling area provides further insight into the gut microbiome divergence observed in the analysed Atlantic cod individuals according to the sampling year, suggesting the establishment of markedly different conditions in the months preceding the December 2021 and January 2024 sampling campaigns. The 2020–2021 period was characterised by colder water, excluding particularly high summer peaks, and, specifically, 2021 by well–oxygenated and generally more oligotrophic waters, whereas the 2023–2024 period showed a clear transition toward warmer, more acidic, and overall, slightly more eutrophic conditions. Our data suggest that, in the coastal ecotype, the greater interannual variation in gut microbiome structure is driven by substantial changes in environmental conditions within coastal habitats. We hypothesise that environmental shifts, particularly increasing water temperature, contributed to the gut microbiome divergence observed between Icelandic cod ecotypes in January 2024 cohort. Rising water temperatures, compounded by the post–pandemic resurgence of anthropogenic activities (Rangel–Buitrago et al. 72, likely precipitated the environmental shifts observed in the study area during the 2023–2024 period, disproportionately impacting coastal ecosystems. Indeed, these fragile environments are thought to be particularly sensitive to the combined impacts of warming and human pressure [2]. In 2024, these combined pressures may have driven detectable shifts in gut microbiome structure within coastal fish populations, including coastal cod. Supporting this hypothesis, the January 2024 coastal cod population was enriched with microbial taxa generally considered as faecal contaminants of anthropogenic origin or linked to anthropogenic activities (*e.g. Streptococcus, Escherichia–Shigella* and *Clostridium*) [18, 48, 59, 83]. However, identifying the drivers underlying the annual divergence in gut microbiome composition between Atlantic cod ecotypes is not straightforward. In addition to ecotype–related factors and contextual environmental drivers, microbiome structure may also be influenced by environmental conditions associated with specific spawning grounds, including the areas where individuals developed during early life stages or those they inhabit during the spawning season [4, 84]. Given the lack of high–resolution tracking data for the sampled individuals, we cannot rule out the potential confounding influence of fjord–specific environmental conditions along the Icelandic coasts that the sampled individuals may have frequented during spawning.

It is worth noting that ecotype designation was based on depth of capture during the feeding season (coastal ecotype, 75 m in both December 2021 and January 2024; frontal ecotype 300 and 304 m in December 2021 and January 2024, respectively). Both ecotypes occur along the coast during the spawning season, but they feed in distinct areas, with resident non–migratory (coastal) cod feeding over the continental shelf in shallow waters, whereas migratory (frontal) individuals move offshore to feed in association with thermal fronts [28, 62]. In this scenario, the observed divergence between ecotypes may be enhanced by their resulting distinct feeding habits [25], indeed, coastal individuals mainly feed on benthic organisms, such as decapods, polychaetes, and gastropods, whereas migratory individuals feeding offshore prey primarily on pelagic fish species [43]. This potential link between ecological factors—including environmental and dietary characteristics—and the host's internal microbial landscape is consistent with the recent characterisation of circulating microbiomes in Atlantic cod by Fronton et al. [27], suggesting that both gut and blood compartments can serve as biological indicators highlighting specific microbial signatures of ecological conditions and environmental pressures.

Moreover, the different ecotypical behaviour has a genetic basis associated with the allele frequency at the *Pantophysin I* (*PanI*) locus [66]. Although the *PanI* locus can be characterised via PCR amplification and Sanger sequencing [61, 66], our ecotype designation was based solely on depth of capture. Although we are confident in our assessment, we cannot entirely rule out the possibility of minor ecotypic misassignments. Such errors should be rare and, therefore, not impact our findings given that all fish were sampled between November and January, outside of the spawning season (March to May, [38], in Icelandic waters and gonads examined were in the “developing” stages.

Our data suggest that gut microbiome divergence between Icelandic cod ecotypes is a dynamic reflection of differential environmental exposure rather than a rigid biological constraint. This plasticity expands upon the findings of Riiser et al. [75], who highlighted how the pronounced divergence between NCC and NEAC ecotypes is associated with contrasting environmental conditions, including differences in depth, temperature, salinity, and dissolved oxygen [20, 21, 56, 85]. Within this context, exposure to local environmental and anthropogenic drivers in coastal habitats can specifically shape the gut microbiome of coastal Icelandic cod, demonstrating how environmental fluctuations can override or accentuate ecotypic signatures even at a small geographical scale.

This tight connection between marine environments and the gut microbiome of Icelandic cod underscores the importance of preserving ecosystem health in the context of global change, both for the conservation of fish populations and for maintaining host–microbiome mutualistic interactions. Concurrently, these observations emphasize the critical need to define the resilience thresholds of the cod–gut microbiome mutualism under the combined weight of shifting climate regimes and intensifying anthropogenic pressures.

## Supplementary Information

Below is the link to the electronic supplementary material.

Supplementary file1 (PDF 1939 KB)

Supplementary file2 (PDF 280 KB)

## Data Availability

High-quality reads from this study were deposited in the European Nucleotide Archive (ENA) under the project accession number PRJEB108649.
