# Supplementary material for: Unraveling the Influence of Behavioural Ecotypes on Fish Gut Microbiome: Focus on the Atlantic cod (Gadus morhua) in Icelandic Waters
Source: Microb Ecol. 2026 Jul 15;89:185. doi: 10.1007/s00248-026-02832-0 (PMC13650084; doi:10.1007/s00248-026-02832-0)
Supplement: Supplementary file 1 [file 248_2026_2832_MOESM1_ESM.pdf]

Unraveling the influence of behavioural ecotypes on fish gut microbiome: focus on the Atlantic cod (*Gadus morhua*) in Icelandic waters

Elena Radaelli [1,2], Giorgia Palladino [1,2], Daniela Leuzzi [1,2], Daniel Scicchitano [1,2], Simone Rampelli [1], Silvia Turroni [1], Haseeb S Randhawa [3,4], Marco Candela [1,2]

[1] Unit of Microbiome Science and Biotechnology, Department of Pharmacy and Biotechnology (FaBit), Alma Mater Studiorum–University of Bologna, Bologna, Italy

[2] Fano Marine Center, the Inter–institute Center for Research on Marine Biodiversity, Resources and Biotechnology, Fano, Italy

[3] Faculty of Life and Environmental Sciences, University of Iceland, Reykjavík, Iceland

[4] New Brunswick Museum, Saint John, New Brunswick, Canada

Supplementary Materials

Supplementary Figures

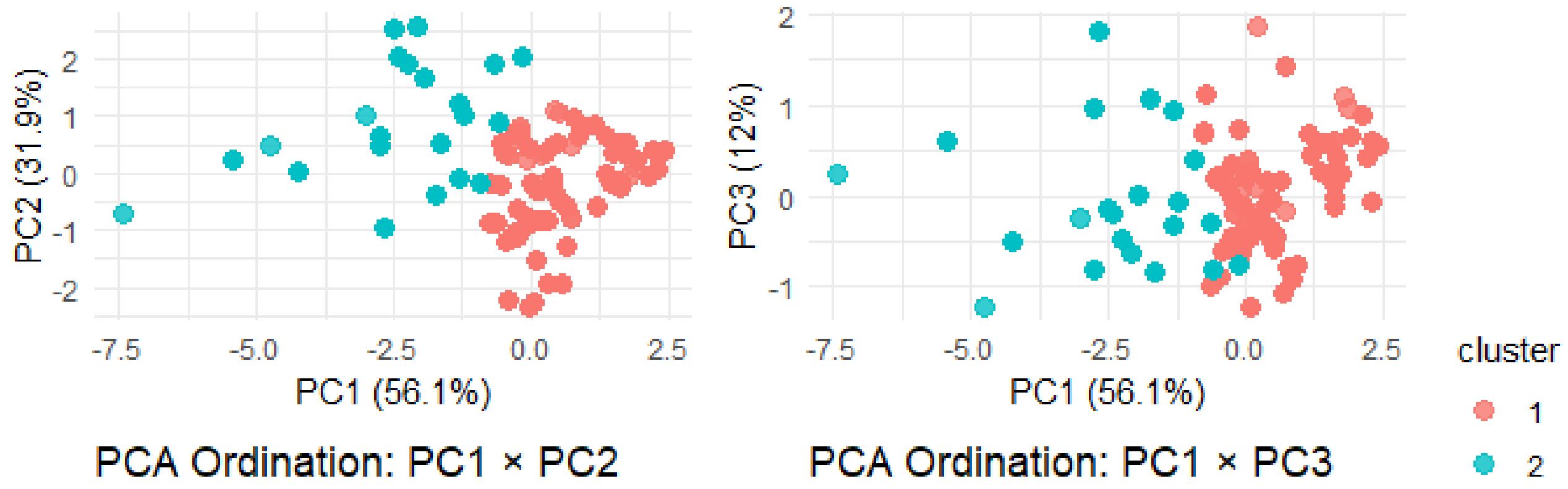

Supplementary Figure S1. PCA ordination plots for stations sampling juvenile cod (2019–2022; not part of this research), by the Marine and Freshwater Research Institute of Iceland, based on environmental variables (sea surface temperature, depth, and sea bottom temperature). Points are coloured by k–means cluster assignment (k = 2). For both species, PC1 represented the dominant depth–temperature gradient. PC2 and PC3 capture secondary temperature–structure variation. The clustering pattern is consistent across species, with a clear separation along PC1 and substantial overlap along PC2 and PC3. The output of this PCA was used to group sampling stations into Area 1 (shallow waters within fjords) and Area 2 (deeper, offshore waters). Figure and caption modified from Win (2026).

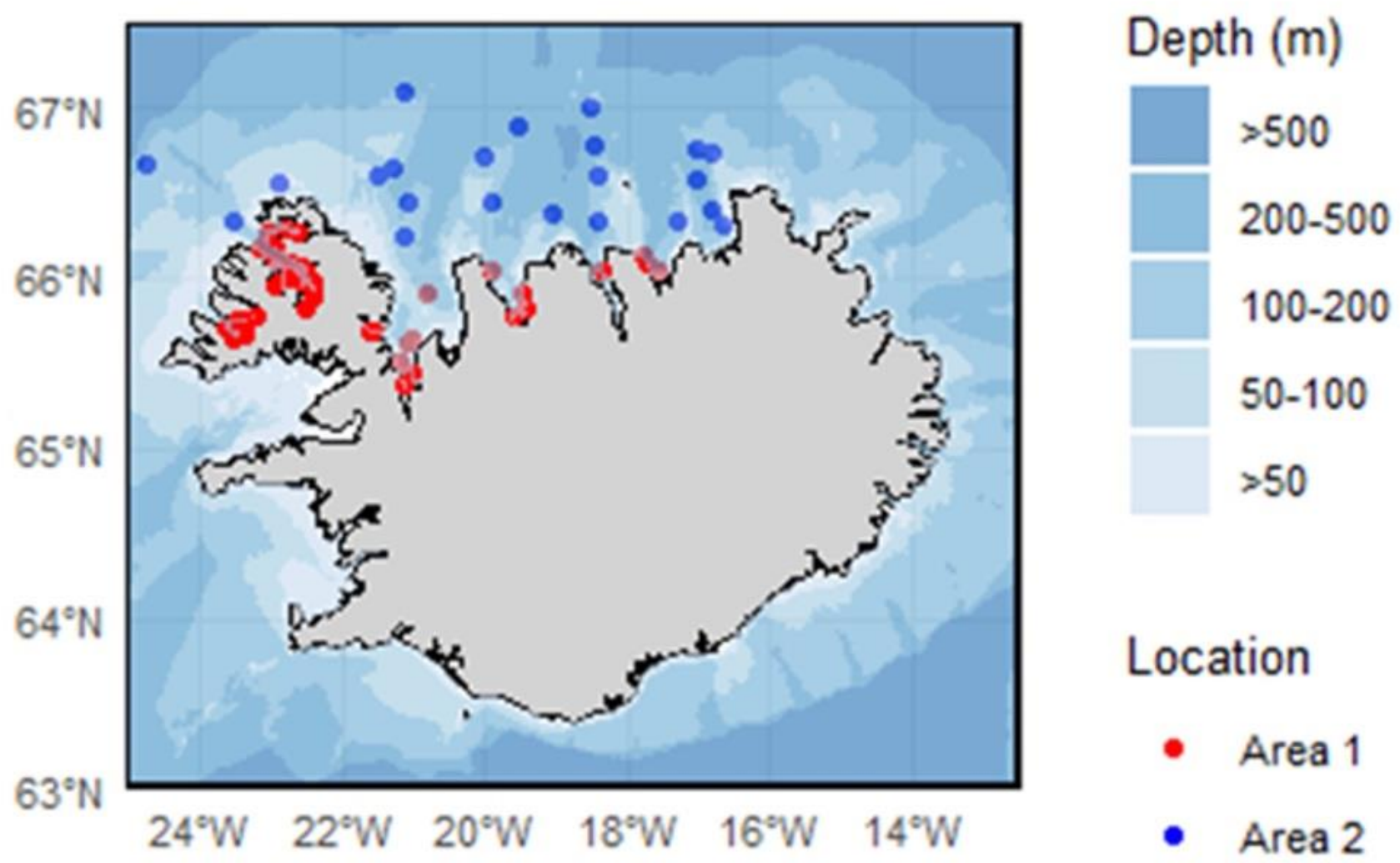

Supplementary Figure S2. Map of Iceland showing the spatial distribution of stations where juvenile cod were sampled (2019–2022; not part of this research) by the Marine and Freshwater Research Institute (Iceland). The areas are based on the PCA output in Supplementary Figure S1, based on environmental variables (sea surface temperature, depth, and sea bottom temperature). The PCA assigned stations into two distinct clusters (by k–means): Area 1 (shallow waters within fjords) and Area 2 (deeper, offshore waters). These were used to designate ecotypes based on sampling locations for our study (see Fig. 1). Figure and caption modified from Win (2026).

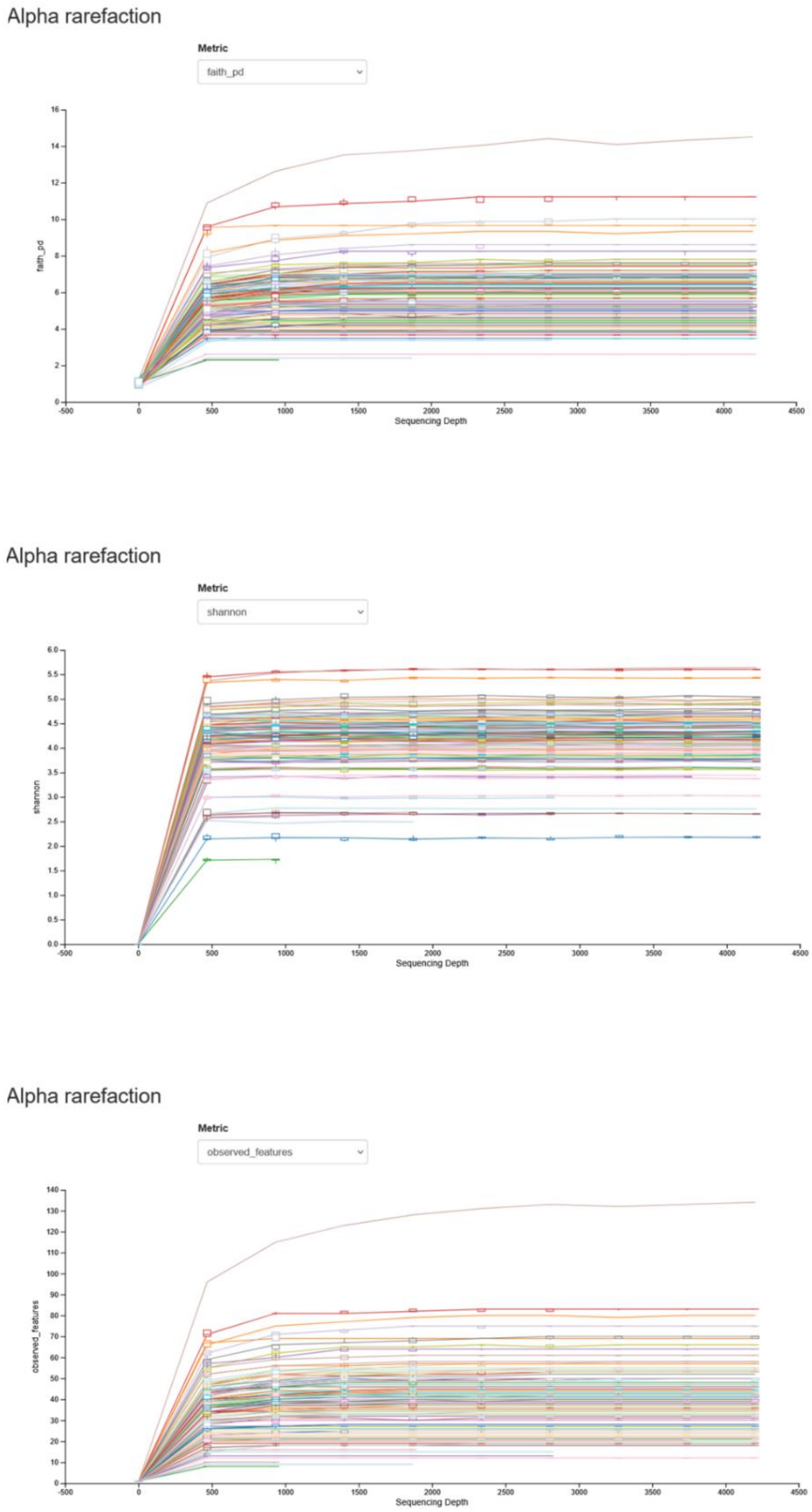

Supplementary Figure S3. Rarefaction curves based on ASV counts for all analysed samples are reported. The saturation of the curves demonstrates that the sequencing depth was sufficient to accurately capture the main microbial diversity. Based on these curves, samples failing to meet the 905 reads threshold were excluded from subsequent downstream analyses.

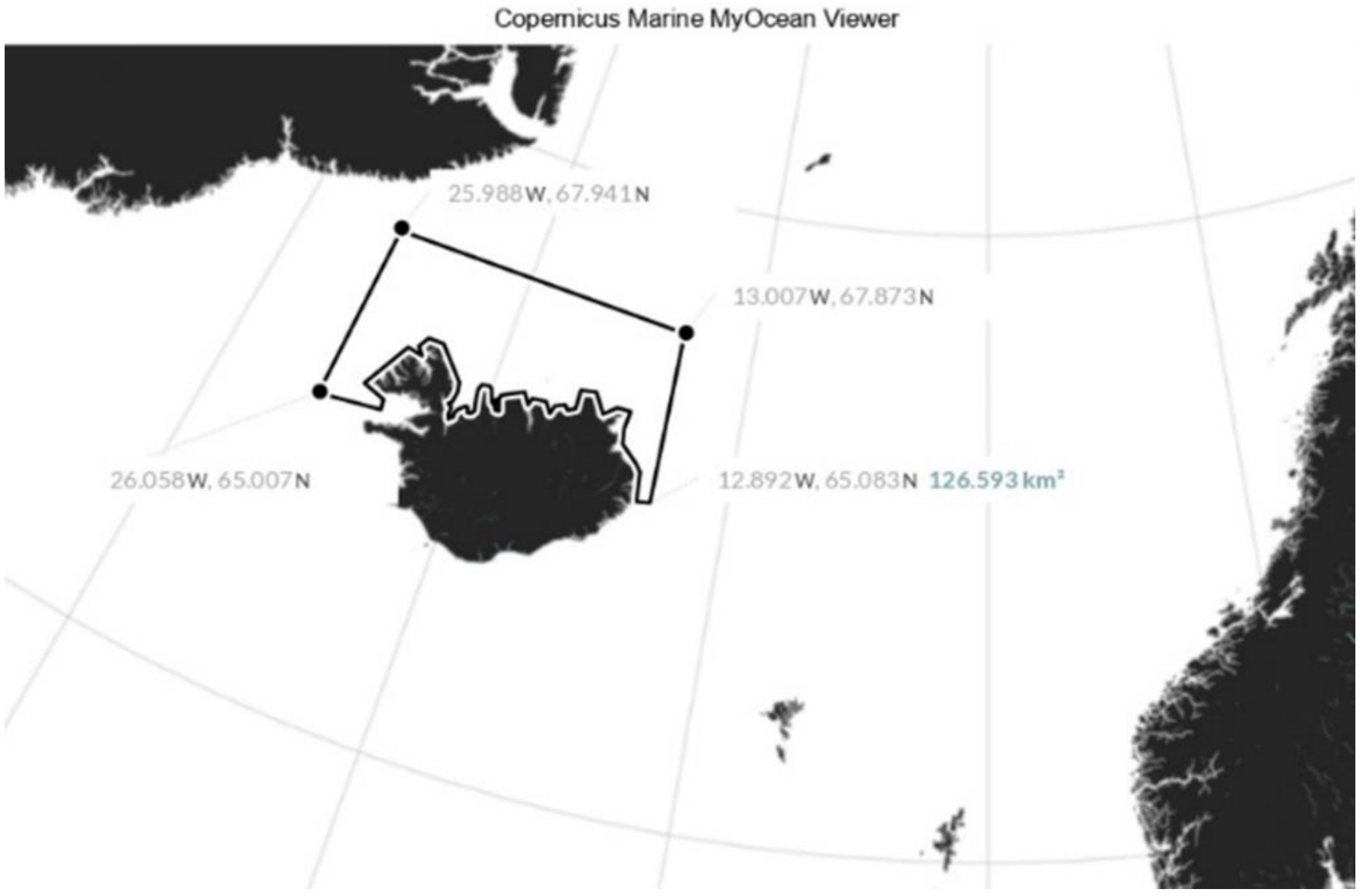

Supplementary Figure S4. Marine area in North Iceland in which environmental parameters were investigated. This area includes sampling points of Atlantic cod individuals collected in December 2021 and January 2024 for this study. Map coordinates and environmental data were downloaded and retrieved from E.U. Copernicus Marine Service Information.

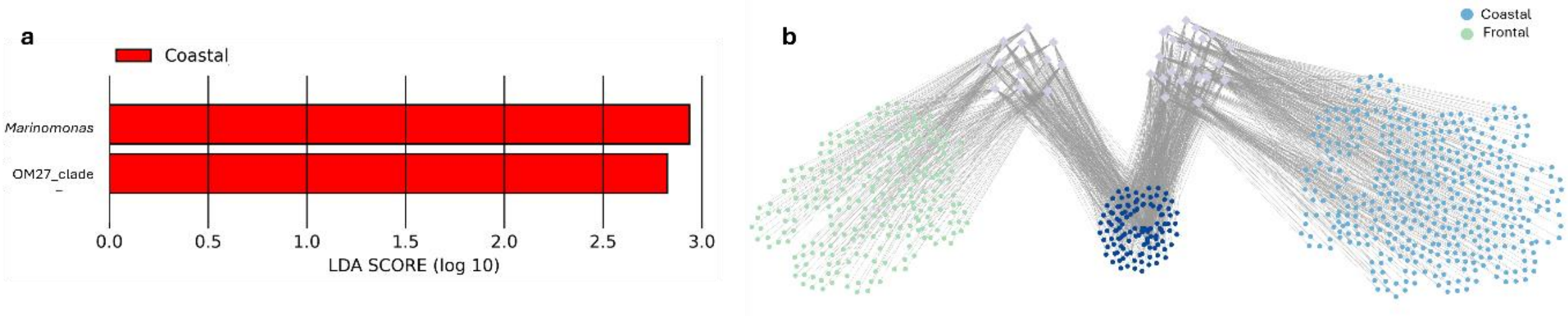

Supplementary Figure S5. Investigation of microbial diversification between ecotypes of cod collected in 2021 **(a)** Lefse analysis performed on gut composition of coastal and frontal ecotypes with LDA score cutoff of 1.5 and a $p < 0.05$. **(b)** OTUs sharing network between ecotypes. OTUs are illustrated with a dot, while samples are represented with a rhombus (purple). Each sample is connected to its OTUs with a line. The network shows OTUs exclusive for coastal (light blue), frontal (green), or shared among the two ecosystems (dark blue).

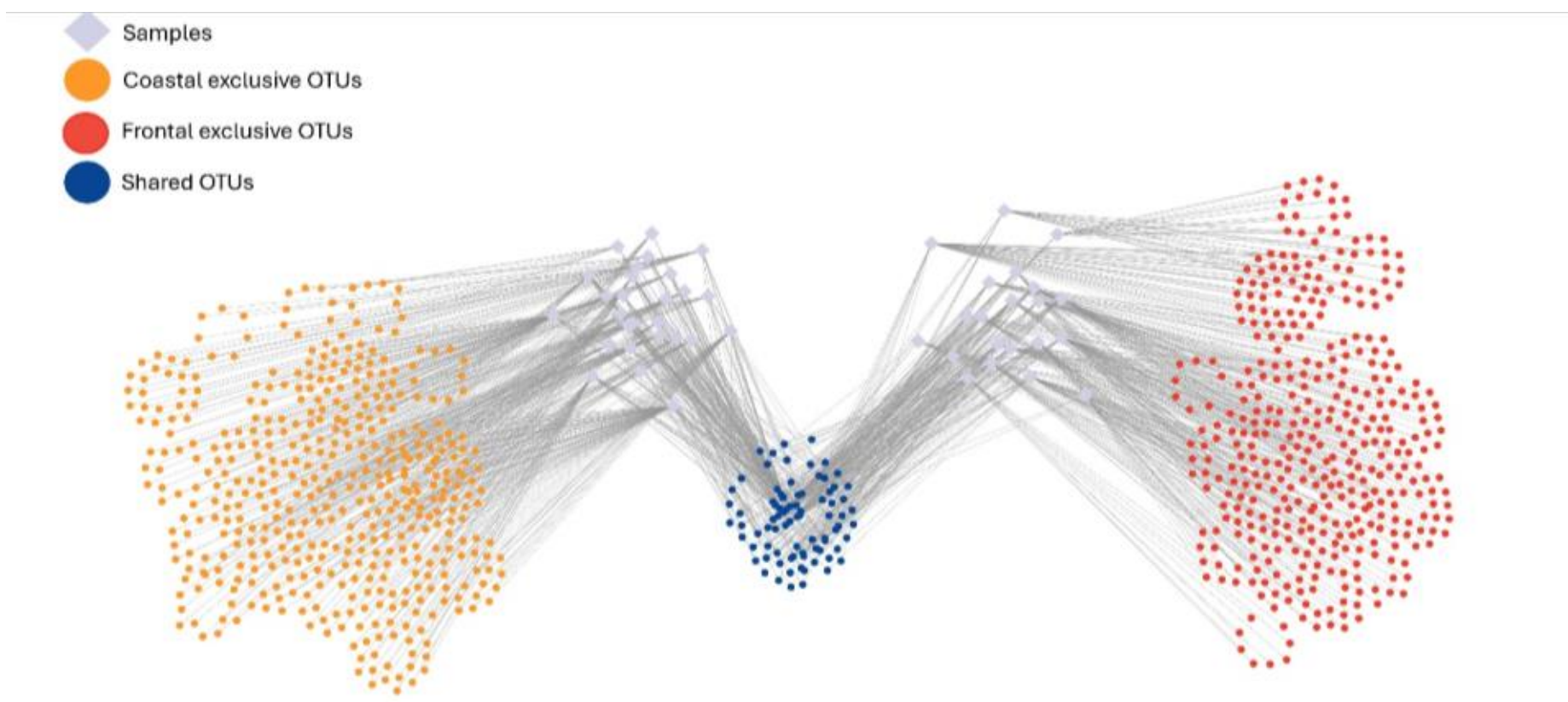

Supplementary Figure S6. OTUs sharing network between ecotypes of cod collected in 2024. OTUs are illustrated with a dot, while samples are represented with a rhombus (purple). Each sample is connected to its OTUs with a line. The network shows OTUs exclusive for coastal (orange), frontal (red), or shared among the two ecosystems (dark blue).

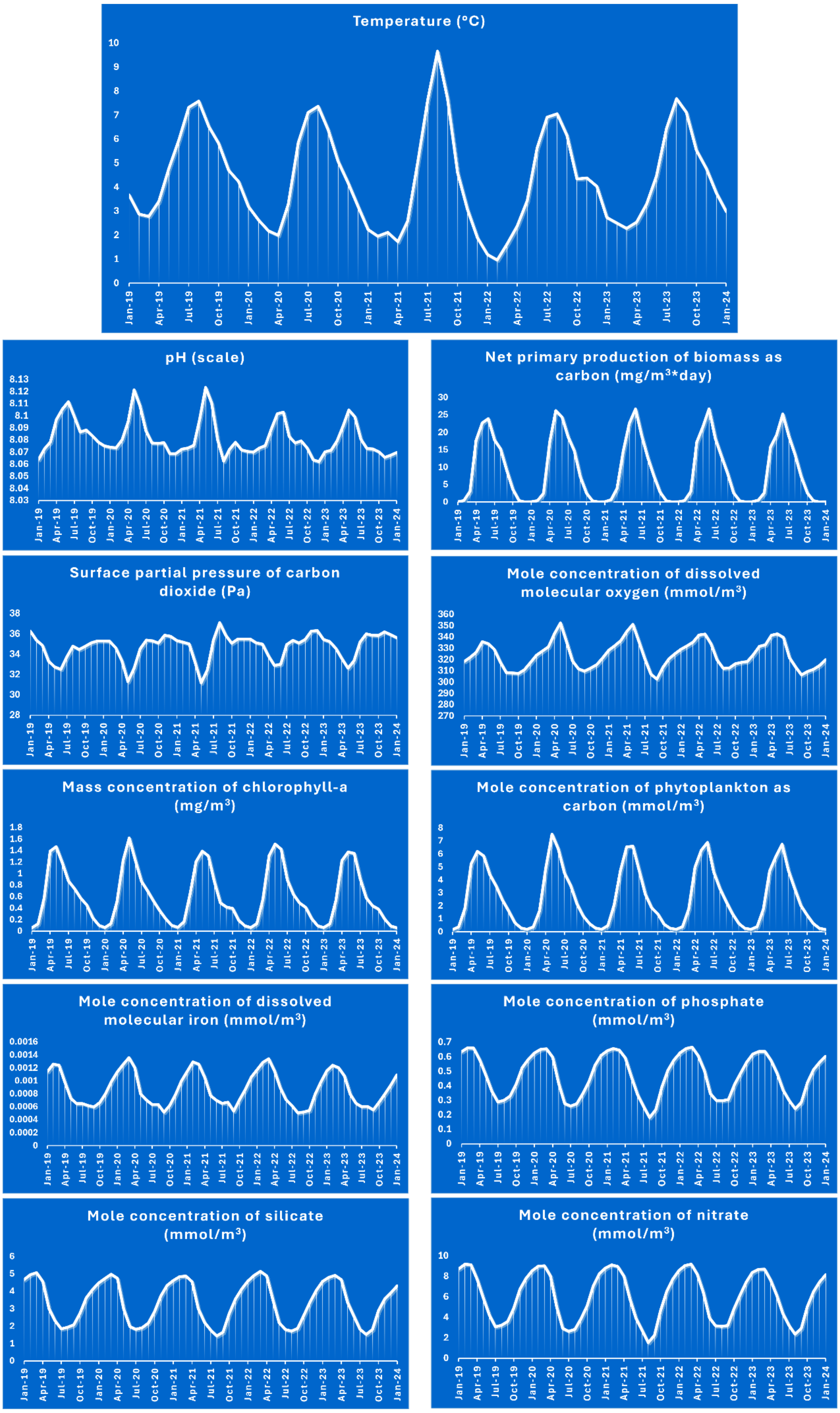

Supplementary Figure S7. Linegraphs of environmental parameters show the trend in environmental parameters month by month throughout the different years. Data values of temperature (°C), pH, net primary production of biomass (mg/m^3*day), carbon dioxide (Pa), dissolved molecular oxygen (mmol/m^3), chlorophyll–a (mg/m^3), phytoplankton (mmol/m^3), dissolved iron (mmol/m^3), phosphate (mmol/m^3), silicate (mmol/m^3), and nitrate (mmol/m^3) were reported for the period from January 2019 to January 2024. Data of water biochemical and physical features were retrieved from E.U. Copernicus Marine Service Information.

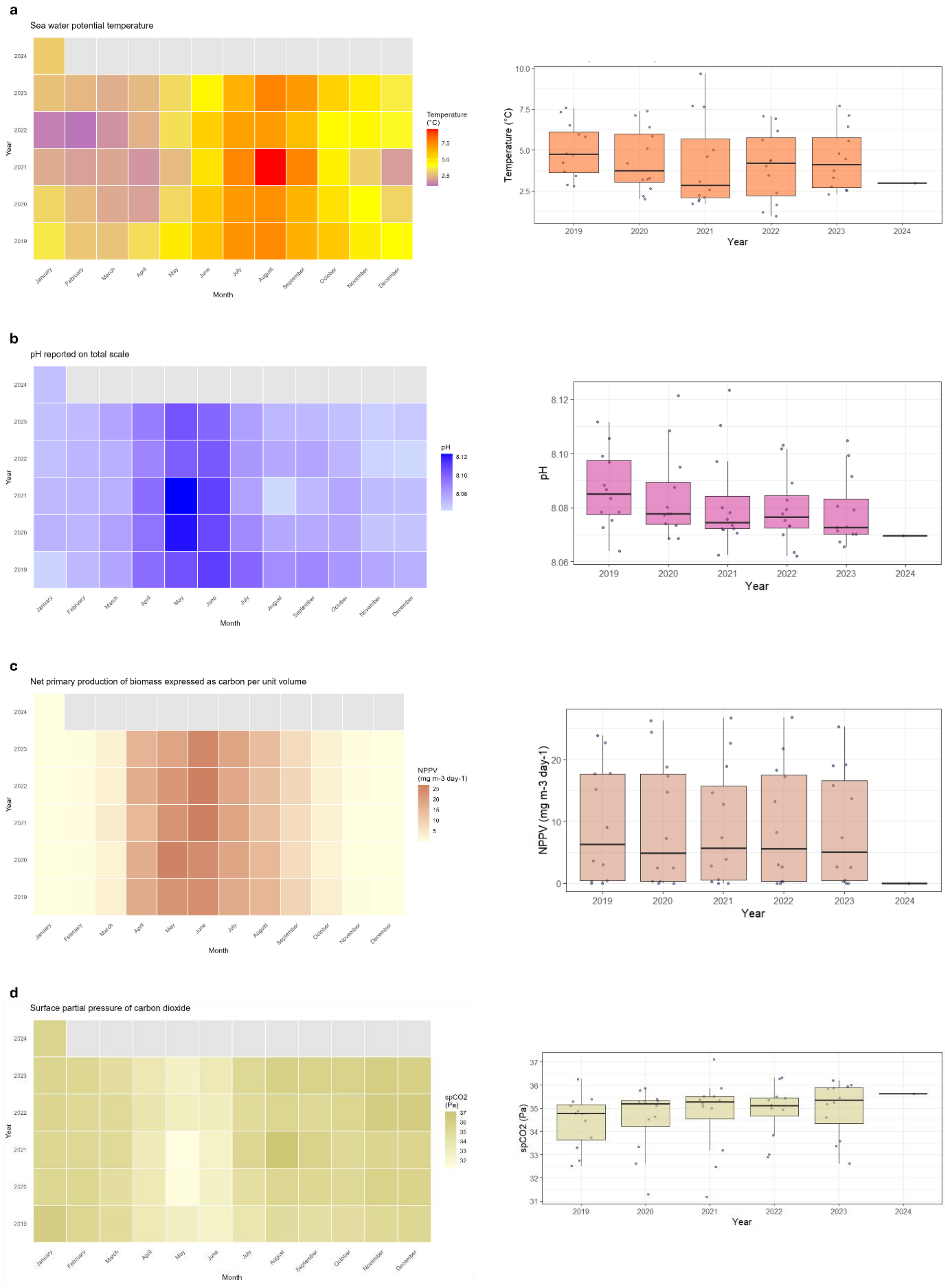
a
Sea water potential temperature
Year
Month
January
February
March
April
May
June
July
August
September
October
November
December
2019
2020
2021
2022
2023
2024
Temperature (°C)
Year
b
pH reported on total scale
pH
Year
c
Net primary production of biomass expressed as carbon per unit volume
NPPV (mg m-3 day-1)
NPPV (mg m-3 day-1)
Year
d
Surface partial pressure of carbon dioxide
spCO2 (Pa)
spCO2 (Pa)
Year

e

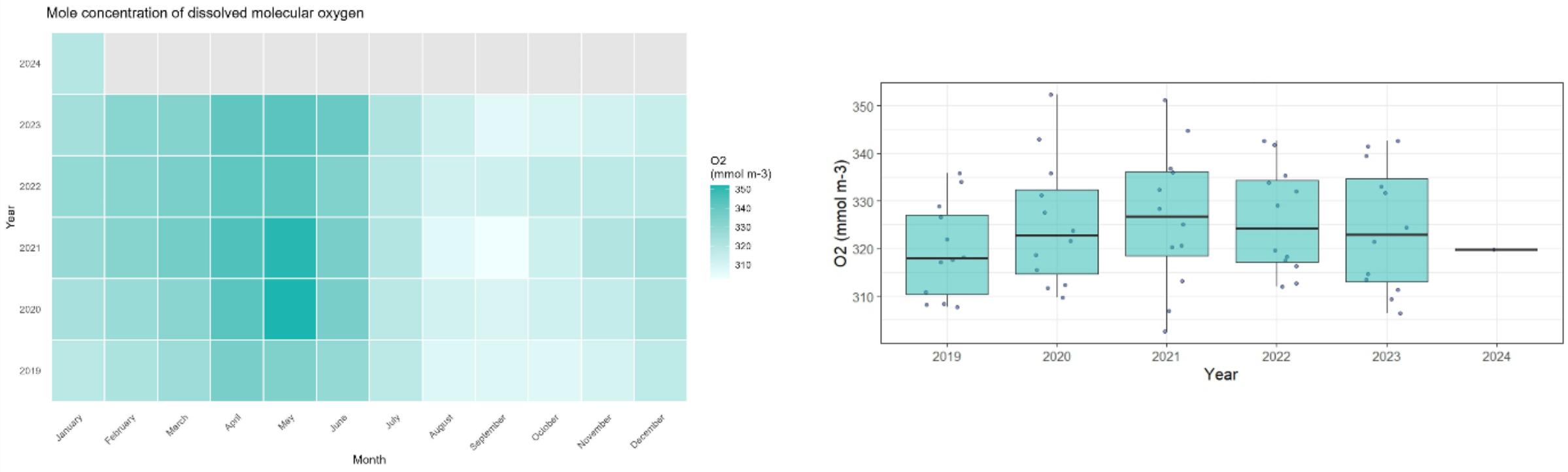
Mole concentration of dissolved molecular oxygen
Year
Month
O2 (mmol m-3)
O2 (mmol m-3)
Year

f

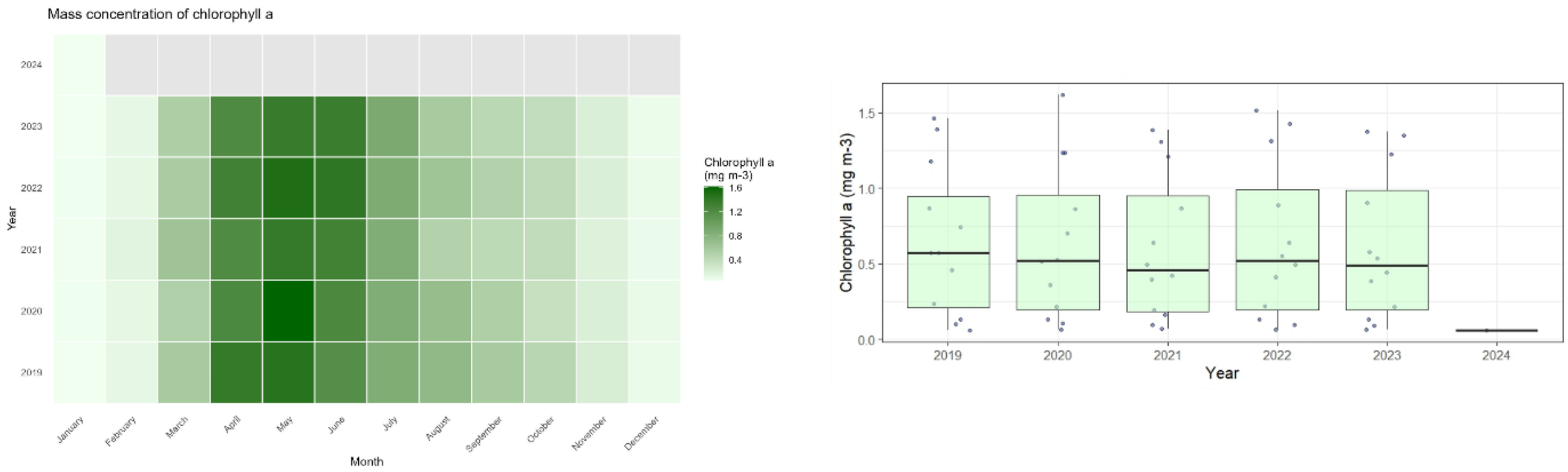
Mass concentration of chlorophyll a
Year
Month
Chlorophyll a (mg m-3)
Chlorophyll a (mg m-3)
Year

g

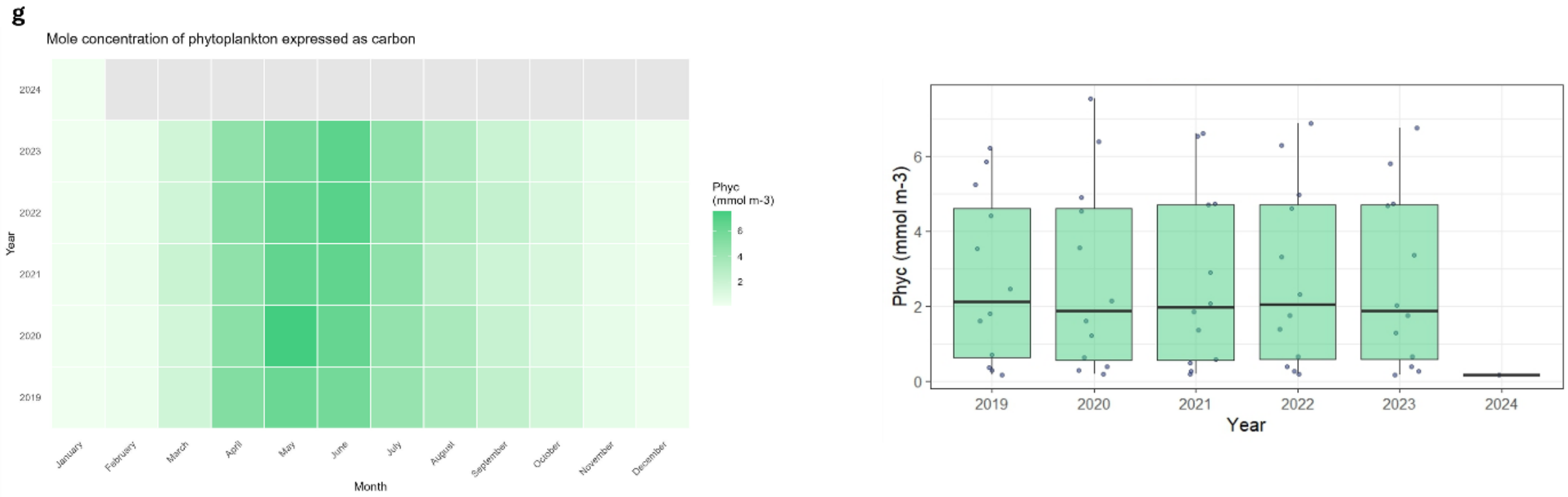
Mole concentration of phytoplankton expressed as carbon
Year
Month
Phyc (mmol m-3)
Phyc (mmol m-3)
Year

h

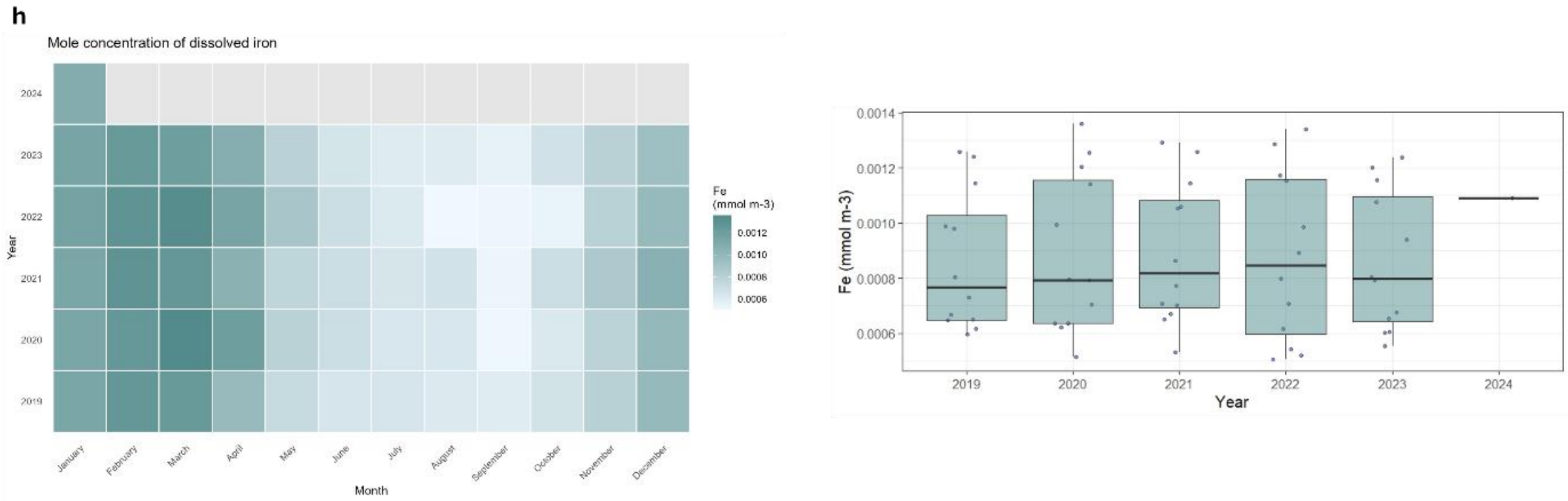
Mole concentration of dissolved iron
Year
Month
Fe (mmol m-3)
Fe (mmol m-3)
Year

i

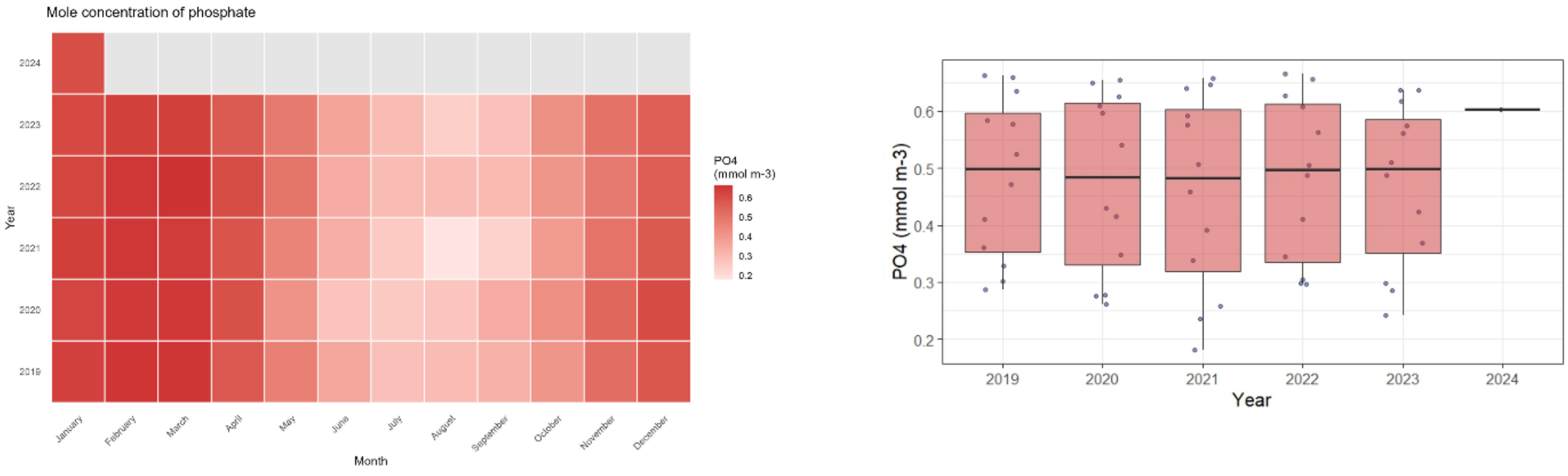

l

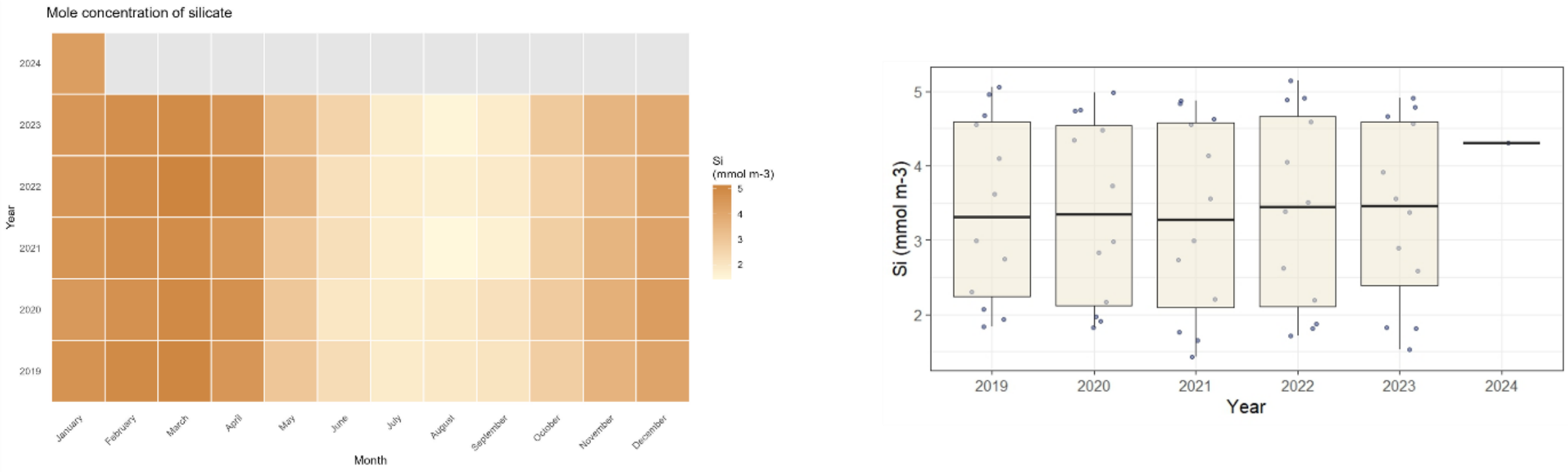

m

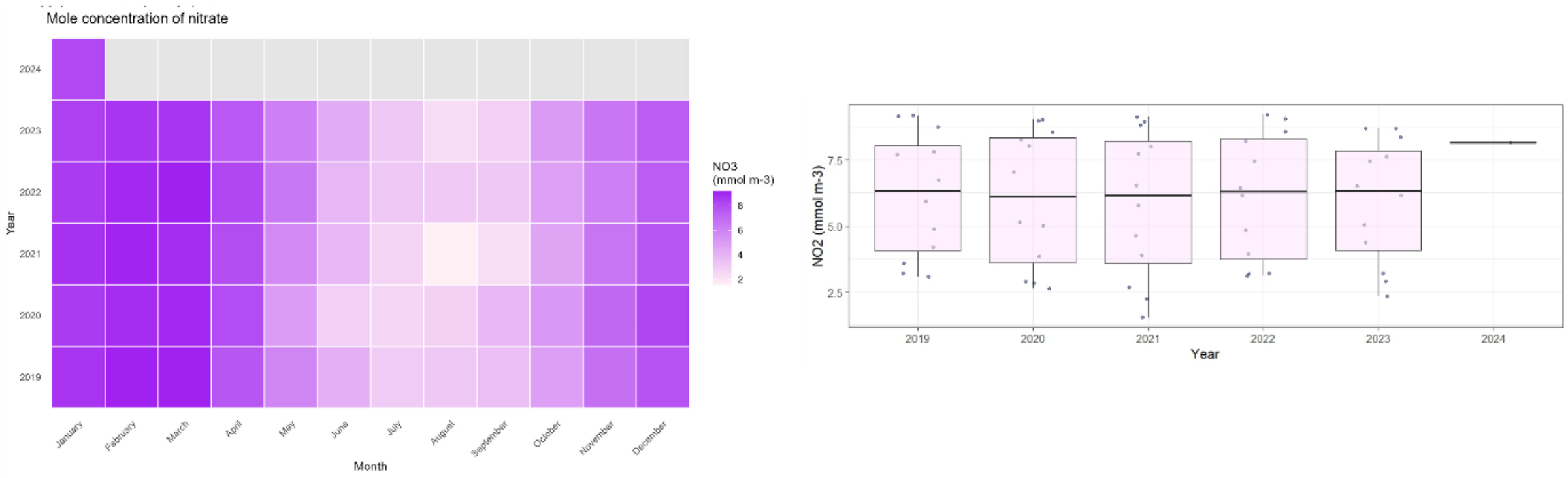

Supplementary Figure S8. Heatmaps, left side, and boxplots, right side, based on monthly mean values of selected environmental parameters in the sampling area of Icelandic cod. Data values of **(a)** temperature (°C), **(b)** pH (scale), **(c)** net primary production of biomass (mg/m^3*day), **(d)** carbon dioxide (Pa), **(e)** dissolved molecular oxygen (mmol/m^3), **(f)** chlorophyll–a (mg/m^3), **(g)** phytoplankton (mmol/m^3), **(h)** dissolved iron (mmol/m^3), **(i)** phosphate (mmol/m^3), **(l)** silicate (mmol/m^3) **(m)** and nitrate (mmol/m^3) were reported for the period from January 2019 to January 2024 (CMEMS). The heatmaps show the trend in environmental parameters month by month throughout the different years with colours reflecting mean monthly values (see legend). The boxplots represent the annual variability of these parameters and their annual median value.

Supplementary Tables

Sample	Ecotype	Date (Sampled)	Depth (m)	Total Length (cm)	Weight (g)
COD014	Coastal	2021–12–06	75	58	1,795
COD015	Coastal	2021–12–06	75	63	2,540
COD017	Coastal	2021–12–06	75	55	1,525
COD018	Coastal	2021–12–06	75	54	1,620
COD019	Coastal	2021–12–07	75	54	1,485
COD020	Coastal	2021–12–07	75	47	950
COD026	Coastal	2021–12–07	75	60	2,090
COD028	Coastal	2021–12–08	75	64	2,740
COD030	Coastal	2021–12–08	75	70	2,980
COD031	Coastal	2021–12–08	75	63	2,270
COD033	Coastal	2021–12–08	75	53	1,270
COD035	Coastal	2021–12–08	75	64	2,590
COD124	Coastal	2024–01–25	75	65	1,830
COD125	Coastal	2024–01–25	75	61	1,748
COD126	Coastal	2024–01–25	75	53	1,476
COD127	Coastal	2024–01–25	75	62	1,776
COD128	Coastal	2024–01–25	75	57	1,552
COD129	Coastal	2024–01–25	75	56	1,298
COD130	Coastal	2024–01–25	75	54	1,384
COD131	Coastal	2024–01–25	75	55	1,450
COD132	Coastal	2024–01–25	75	64	1,808
COD133	Coastal	2024–01–25	75	63	1,850
COD134	Coastal	2024–01–25	75	59	1,506
COD135	Coastal	2024–01–25	75	54	1,126
COD136	Coastal	2024–01–25	75	56	1,426
COD137	Coastal	2024–01–25	75	60	1,644
COD138	Coastal	2024–01–25	75	57	1,388
COD139	Coastal	2024–01–25	75	57	1,428
COD140	Coastal	2024–01–25	75	59	1,738
COD141	Coastal	2024–01–25	75	58	1,476
COD142	Coastal	2024–01–25	75	53	1,122
COD143	Coastal	2024–01–25	75	61	1,546
COD144	Coastal	2024–01–25	75	60	1,514
COD145	Coastal	2024–01–25	75	58	1,762
COD146	Coastal	2024–01–25	75	63	1,908
COD147	Coastal	2024–01–25	75	60	1,678
COD148	Coastal	2024–01–25	75	59	1,612
COD149	Coastal	2024–01–25	75	62	1,700
COD150	Coastal	2024–01–25	75	58	1,300
COD021	Frontal	2021–12–07	300	89	4,910
COD022	Frontal	2021–12–07	300	74	2,940
COD023	Frontal	2021–12–07	300	58	1,640
COD024	Frontal	2021–12–07	300	83	4,810
COD025	Frontal	2021–12–07	300	83	4,520
COD036	Frontal	2021–12–08	300	71	3,430
COD038	Frontal	2021–12–08	300	74	3,010
COD039	Frontal	2021–12–08	300	76	2,940
COD040	Frontal	2021–12–08	300	67	2,590
COD042	Frontal	2021–12–08	300	62	2,050
COD043	Frontal	2021–12–08	300	77	3,910
COD044	Frontal	2021–12–09	300	60	2,490
COD046	Frontal	2021–12–09	300	85	5,800
COD047	Frontal	2021–12–09	300	74	3,550

COD048	Frontal	2021–12–09	300	92	6,220
COD051	Frontal	2021–12–09	300	82	4,010
COD053	Frontal	2021–12–09	300	73	3,120
COD055	Frontal	2021–12–09	300	50	1,010
COD056	Frontal	2021–12–09	300	79	3,160
COD057	Frontal	2021–12–09	300	78	3,750
COD058	Frontal	2021–12–09	300	83	4,520
COD059	Frontal	2021–12–09	300	76	3,430
COD101	Frontal	2024–01–16	304	78	3,186
COD102	Frontal	2024–01–16	304	50	996
COD103	Frontal	2024–01–16	304	55	1,390
COD104	Frontal	2024–01–16	304	86	5,344
COD105	Frontal	2024–01–17	304	81	4,810
COD106	Frontal	2024–01–17	304	75	3,738
COD107	Frontal	2024–01–17	304	74	3,100
COD108	Frontal	2024–01–17	304	70	2,930
COD109	Frontal	2024–01–17	304	76	3,894
COD110	Frontal	2024–01–17	304	69	2,950
COD111	Frontal	2024–01–18	304	74	3,516
COD112	Frontal	2024–01–18	304	77	3,854
COD113	Frontal	2024–01–18	304	74	3,114
COD114	Frontal	2024–01–18	304	71	3,054
COD115	Frontal	2024–01–19	304	76	3,904
COD116	Frontal	2024–01–19	304	53	1,504
COD117	Frontal	2024–01–19	304	62	2,050
COD119	Frontal	2024–01–19	304	58	1,466
COD120	Frontal	2024–01–19	304	59	1,716
COD121	Frontal	2024–01–19	304	53	1,232
COD122	Frontal	2024–01–19	304	65	2,174
COD123	Frontal	2024–01–19	304	53	1,294

Supplementary Table S1. Table reporting metadata of Atlantic cod (*Gadus morhua*) individuals such as ecotype, sampling date and depth, total length (cm), and weight (g).

**Mean total length (cm)**		
	Coastal	Frontal
December 2021	59 ± 6	75 ± 10
January 2024	59 ± 3	63 ± 11
Overall (2021–2024)	59 ± 4	71 ± 11
**Mean weight (g)**		
	Coastal	Frontal
December 2021	1,988 ± 642	3,537 ± 1,266
January 2024	1,557 ± 213	2,783 ± 1,223
Overall (2021–2024)	1,690 ± 437	3,160 ± 1,288

Supplementary Table S2. Table reporting mean and standard deviation values of total length (cm) and weight (g) of Icelandic cod collected in December 2021 and January 2024. Values are reported by annual ecotype and for combined ecotype data across both sampling periods.

Coastal Ecotype			Frontal Ecotype		
**Weighted UniFrac**			**Weighted UniFrac**		
Variabile	Rho_MDS1	Pval_MDS1	Variabile	Rho_MDS1	Pval_MDS1
Temperature	0.602221359	4.99E-05	Temperature	-0.009872481	0.952435131
Chlorophyll *a*	-0.602221359	4.99E-05	Chlorophyll *a*	0.009872481	0.952435131
Dissolved Iron	0.602221359	4.99E-05	Dissolved Iron	-0.009872481	0.952435131
Dissolved Oxygen	-0.602221359	4.99E-05	Dissolved Oxygen	0.009872481	0.952435131
Pressure of Carbon Dioxide	0.602221359	4.99E-05	Pressure of Carbon Dioxide	-0.009872481	0.952435131
Nitrate	-0.602221359	4.99E-05	Nitrate	0.009872481	0.952435131
Phosphate	0.602221359	4.99E-05	Phosphate	-0.009872481	0.952435131
Phytoplankton	-0.602221359	4.99E-05	Phytoplankton	0.009872481	0.952435131
Silicate	-0.602221359	4.99E-05	Silicate	0.009872481	0.952435131
Net Primary Production	-0.602221359	4.99E-05	Net Primary Production	0.009872481	0.952435131
pH	0.602221359	4.99E-05	pH	-0.009872481	0.952435131
**Unweighted UniFrac**			**Unweighted UniFrac**		
Variabile	Rho_MDS1	Pval_MDS1	Variabile	Rho_MDS1	Pval_MDS1
Temperature	0.631838803	1.60E-05	Temperature	-0.207322107	0.20536474
Chlorophyll *a*	-0.631838803	1.60E-05	Chlorophyll *a*	0.207322107	0.20536474
Dissolved Iron	0.631838803	1.60E-05	Dissolved Iron	-0.207322107	0.20536474
Dissolved Oxygen	-0.631838803	1.60E-05	Dissolved Oxygen	0.207322107	0.20536474
Pressure of Carbon Dioxide	0.631838803	1.60E-05	Pressure of Carbon Dioxide	-0.207322107	0.20536474
Nitrate	-0.631838803	1.60E-05	Nitrate	0.207322107	0.20536474
Phosphate	0.631838803	1.60E-05	Phosphate	-0.207322107	0.20536474
Phytoplankton	-0.631838803	1.60E-05	Phytoplankton	0.207322107	0.20536474
Silicate	-0.631838803	1.60E-05	Silicate	0.207322107	0.20536474
Net Primary Production	-0.631838803	1.60E-05	Net Primary Production	0.207322107	0.20536474
pH	0.631838803	1.60E-05	pH	-0.207322107	0.20536474

Supplementary Table S3. Spearman's rank correlation coefficients (rho) and corresponding p–values between 11 environmental parameters and sample ordination scores along the MDS1 axis, calculated for both unweighted and weighted UniFrac PCoA (Fig. 6c–d–e–f). For the coastal ecotype, all correlations were significant ($p < 0.001$), whereas no significant relationships with the MDS1 axis were detected for the frontal ecotype ($p > 0.05$).
