# Supplementary material for: Unraveling the Influence of Behavioural Ecotypes on Fish Gut Microbiome: Focus on the Atlantic cod (Gadus morhua) in Icelandic Waters
Source: Microb Ecol. 2026 Jul 15;89:185. doi: 10.1007/s00248-026-02832-0 (PMC13650084; doi:10.1007/s00248-026-02832-0)
Supplement: Supplementary file 2 [file 248_2026_2832_MOESM2_ESM.pdf]

Unraveling the influence of behavioural ecotypes on fish gut microbiome: focus on the Atlantic cod (Gadus morhua) in Icelandic waters

Elena Radaelli [1,2], Giorgia Palladino [1,2], Daniela Leuzzi [1,2], Daniel Scicchitano [1,2], Simone Rampelli [1], Silvia Turroni [1], Haseeb S Randhawa [3,4], Marco Candela [1,2]

[1] Unit of Microbiome Science and Biotechnology, Department of Pharmacy and Biotechnology (FaBit), Alma Mater Studiorum– University of Bologna, Bologna, Italy

[2] Fano Marine Center, the Inter–institute Center for Research on Marine Biodiversity, Resources and Biotechnology, Fano, Italy

[3] Faculty of Life and Environmental Sciences, University of Iceland, Reykjavík, Iceland

[4] New Brunswick Museum, Saint John, New Brunswick, Canada

Supplementary Information–Analyses on environmental parameters retrieved by E.U. Copernicus Marine Service Information (CMEMS)

Temperature (°C)				
	2019	2020	2021	2022
**2020**	*0.01858	–	–	–
**2021**	*0.00342	1.00000	–	–
**2022**	*0.00055	0.89832	1.00000	–
**2023**	0.17966	1.00000	0.65397	0.21975

Dissolved molecular oxygen (mmol/m³)				
	2019	2020	2021	2022
**2020**	*0.01256	–	–	–
**2021**	*0.00010	0.57821	–	–
**2022**	*0.00045	0.82342	1.00000	–
**2023**	*0.01736	1.00000	0.50648	0.82342

pH (scale)				
	2019	2020	2021	2022
**2020**	1.0000	–	–	–
**2021**	0.6822	1.0000	–	–
**2022**	*0.0057	*0.0177	0.1495	–
**2023**	*0.0057	*0.0177	0.1495	1.000

Table 1. Outcomes of the Conover–Iman *post–hoc* test after the Friedman rank sum test considering temperature (*C), dissolved molecular oxygen (mmol/m3) and pH (scale) as environmental variables significantly changing across the years 2019–2023. Annual couples having significant differences ($p < 0.05$) are marked with an asterisk.

Temperature (°C)	
**Year**	**Grouping letters**
2019	a
2020	b
2021	b
2022	b
2023	ab

Dissolved molecular oxygen (mmol/m³)	
**Year**	**Grouping letters**
2019	a
2020	b
2021	b
2022	b
2023	b

pH (scale)	
**Year**	**Grouping letters**
2019	a
2020	a
2021	ab
2022	b
2023	b

Table 2. The compact letter display (CLD) method test between years considering as parameters: temperature (°C), dissolved molecular oxygen (mmol/m³), and pH (scale). Years with different letters indicate a significant divergence based on Conover–Iman post–hoc test, $p < 0.05$, while years with at least one letter in common are not significantly different.

To deepen the investigation of interannual differences in environmental parameters (2019–2023), statistical analyses were carried out using the non–parametric Friedman test (Friedman M, 1937). This approach was selected due to the small amount of data and to account for monthly seasonality, treating months as blocking factors. When the p–value was significant, the Conover–Iman *post–hoc* test (Conover & Iman, 1979) was performed for pairwise comparisons.

To control for Type I error inflation, p–values were adjusted using the Holm–Bonferroni method (Holm S, 1979). The Compact Letter Display (CLD) (Gegzna, 2025) was used in order to show the changing trends along years according to the p–values retrieved by Conover–Iman test.

Considering the impact of seasonality across years, the Friedman rank sum test was then performed on years from 2019 to 2023 using R software as additional non–parametric statistical test incorporating seasonal trends. Whenever the Friedman test indicated a significant global difference ($p < 0.05$), the Conover–Iman *post–hoc* test was used to identify subtle differences between annual pairs. Using the Friedman test, significant p–values for sea water temperature ($p = 0.001876$), oxygen ($p = 0.0006173$), and pH ($p = 0.001616$) parameters were detected. Outcomes of the Conover–Iman *post–hoc* test considering temperature, oxygen and pH parameters (Tab. 1) highlight that 2019 is the year that differs most significantly from the following years considering the three parameters. The compact letter display (CLD) method (Tab. 2) was performed in order to more clearly show changing trends of environmental parameters across the years, since the representation of the statistical separation between the studied years is characterized by different letters in case of significant shifts, by same letters in case of statistical homogeneity, and by more than one overlapped letter in case of transitional states. Looking at these results and integrating them with the annual variability of temperature, oxygen and pH parameters shown in the heatmaps and boxplots of the article (Supplementary Fig. S8), the visible trends of annual variability for these environmental parameters are partially supported by statistical analyses. The temperature appears to be stable between 2020, 2021, and 2022 compared to 2019, which stands out as a remarkably warm year. The 2023 occupies an intermediate state, showing a slight increase in temperature compared to the previous three years. Considering the dissolved molecular oxygen, the 2019 differs from the others, despite the clear variability trend of the median value, which peaks in 2021 and begins to decline again the following year. Finally, the pH trend shows significant variability between the 2019–2020 and 2022–2023 groups, confirming an acidification trend with a significant impact already after a period of two years.

Literature cited

1. Conover, W. J., & Iman, R. L. (1979). *On multiple–comparisons procedures* (Tech. Rep. LA–7677–MS). Los Alamos Scientific Laboratory. https://doi.org/10.2172/6057803

2. Friedman, M. (1937). The use of ranks to avoid the assumption of normality implicit in the analysis of variance. *Journal of the American Statistical Association*, *32*(200), 675–701. https://doi.org/10.2307/2279372

3. Gegzna, V. (2025). *cld: Create Compact Letter Display (CLD) for Statistical Comparisons* (Versione 0.0.1) [Software]. https://github.com/gegznav/cld

4. Holm, S. (1979). A simple sequentially rejective multiple test procedure. *Scandinavian Journal of Statistics*, *6*(2), 65–70. [suspicious link removed]
